# Unraveling the Bone–Brain Communication Network

**DOI:** 10.3390/biology14091279

**Published:** 2025-09-17

**Authors:** Surajit Hansda, Hiranmoy Das

**Affiliations:** Department of Pharmaceutical Sciences, Jerry H. Hodge School of Pharmacy, Texas Tech University Health Sciences Center, ARB Suite 2116, 1406 South Coulter Street, Amarillo, TX 79106, USA; shansda@ttuhsc.edu

**Keywords:** brain, bone, aging, molecular signaling, inflammation

## Abstract

The bone and brain, though distinct in structure and function, share remarkable physical, molecular, and developmental similarities. Emerging evidence reveals dynamic bidirectional crosstalk between these systems mediated by hormones, cytokines, extracellular vesicles (EVs), and neural signals. Bone-derived factors such as osteocalcin (OCN), lipocalin-2, and fibroblast growth factor (FGF) 23 influence cognitive functions, mood, and neurogenesis, while brain- and nerve-derived mediators, including leptin, serotonin, and sympathetic signals, modulate bone remodeling. Inflammation and aging disrupt this communication, contributing to cognitive decline, osteoporosis, and other age-related disorders. Stem cells and EVs have also been implicated as mediators in this axis, offering insights into regenerative strategies. Molecular signaling pathways and transcriptional regulators, such as Wnt/β-catenin, leptin, receptor activator of nuclear factor kappa-B ligand (RANKL), sclerostin (SOST), and nuclear factor kappa-B (NF-κB), play critical roles in maintaining bone–brain homeostasis. Additionally, shared biomarkers and pathological links between neurodegeneration and bone loss suggest new diagnostic and therapeutic opportunities. Studies support this inter-organ communication, yet further mechanistic and translational research is needed. This review highlights the molecular basis of bone–brain crosstalk, emphasizing inflammation, aging, and regulatory pathways, with a focus on future directions in biomarker discovery and therapeutic targeting. Understanding this crosstalk may help in early diagnosis and dual-targeted interventions for both bone and brain disorders.

## Simple Summary

Although the brain and bones seem unrelated, new research shows they communicate in surprising ways. Chemicals released by bones can affect brain functions like memory and mood, while the brain sends signals that influence bone growth and strength. This two-way connection helps maintain the health of both systems. As we age or face chronic inflammation, this communication can break down, leading to problems like weaker bones or memory decline. Researchers have also found that stem cells and tiny messengers from cells may help repair damage in both the brain and bones. By understanding how these systems interact, scientists hope to discover new ways to detect diseases early and treat conditions like osteoporosis and neurodegenerative disorders at the same time. This article could lead to the design of future therapies that protect both the mind and the body. The review highlights how this bone–brain “crosstalk” opens the door to new, more effective treatments.

## 1. Introduction

The conventional boundaries of organ systems are rapidly being redrawn by an evolving apprehension of inter-organ communication. Long perceived as functionally isolated entities, the brain and bone are now recognized as critical co-regulators of homeostasis, with dynamic crosstalk involving neural, endocrine, immune, and metabolic pathways. While the brain orchestrates systemic control through central and peripheral signaling, bone, far beyond its mechanical and structural functions, has emerged as a metabolically active organ capable of endocrine and paracrine influence on distant tissues, including the central nervous system (CNS) [1,2].

The brain exerts substantial control over bone remodeling via hypothalamic neuroendocrine signals and autonomic innervation, primarily through the sympathetic nervous system (SNS). Neurotransmitters and hormones such as norepinephrine, serotonin, and leptin regulate the balance between osteoblast-mediated bone formation and osteoclast-mediated resorption [3,4]. On the other hand, the skeleton secretes several bone-derived factors, often termed osteokines, including osteocalcin (OCN), fibroblast growth factor 23 (FGF23), lipocalin-2 (LCN2), and sclerostin, which can cross the blood–brain barrier (BBB) and exert effects on neurogenesis, neurotransmitter synthesis, cognitive functions, and mood regulation [5,6]. This emerging paradigm of skeletal endocrinology has redefined bone as a central player in systemic health.

Notably, the interplay between bone and brain is highly influenced by inflammation and aging. Inflammatory mediators such as tumor necrosis factor-alpha (TNF-α), interleukin-1 beta (IL-1β), and interleukin-6 (IL-6), which are elevated in both osteoporosis and neurodegenerative diseases, serve as common mechanistic links [7,8,9,10]. Aging exacerbates these effects through the accumulation of senescent cells, dysregulation of immune responses, and decline in anabolic hormones, thereby creating a pro-inflammatory microenvironment that disrupts bone and brain homeostasis [11,12]. Cellular aging pathways involving sirtuin 1 (SIRT1), mammalian target of rapamycin (mTOR), and nuclear factor kappa-light-chain-enhancer of activated B cells (NF-κB) appear to orchestrate shared transcriptional programs in both bone and brain tissues [13,14,15]. This convergence of inflammatory and epigenetic regulation represents a compelling target landscape for intervention.

Emerging studies in transcriptomics and epigenomics have further illuminated the shared regulatory architecture of the brain and bone. Single-cell RNA sequencing has revealed overlapping gene expression signatures between osteocytes and neurons, particularly in genes associated with synaptic signaling, vesicle transport, and mitochondrial function [16,17]. Transcriptional regulators such as runt-related transcription factor 2 (RUNX2), cyclic adenosine monophosphate responsive element binding protein (CREB), forkhead box O3 (FOXO3), and myocyte enhancer factor 2 (MEF2) have been implicated in both osteogenesis and neuroplasticity, suggesting a conserved regulatory logic governing differentiation, stress response, and cell survival. Transcriptional dysregulation is increasingly being recognized as a convergent mechanism underlying both cognitive decline and skeletal fragility in aging populations [18,19,20].

Mechanical stimulation and skeletal interoception also form an essential dimension of bone–brain crosstalk. The skeleton acts as a sensory organ, translating mechanical signals into biochemical cues via osteocytes, which in turn influence neural circuits involved in posture, proprioception, and energy balance [21,22]. Bone cells respond to mechanical stimulation by releasing signaling molecules, such as prostaglandin E_2_ (PGE_2_), nitric oxide (NO), and ATP. PGE_2_ activates prostaglandin E2 receptor EP4 subtype (EP4) on sensory nerves, transmitting signals that influence bone remodeling (sympathetic activity) [23,24]. Meanwhile, ATP released from mechanically stimulated osteoblasts and osteocytes propagates intercellular calcium waves through purinergic signaling, which facilitates local mechanotransduction; however, direct nerve activation remains to be established [25,26]. Moreover, alterations in mechanical loading due to aging, sedentary behavior, or spaceflight can disrupt this mechano-transduction loop, contributing to both bone loss and cognitive dysfunction.

There is a substantial clinical relevance of the bone–brain crosstalk. Osteoporosis and Alzheimer’s disease (AD), for instance, frequently co-occur, with shared risk factors such as chronic inflammation, mitochondrial dysfunction, and hormonal imbalances. Hip fractures in elderly patients are often followed by rapid cognitive decline and increased mortality, pointing to a deep-rooted physiological connection between the brain and bone [27,28,29]. However, therapeutic interventions have largely remained siloed within specialty disciplines, failing to capitalize on this systemic interdependence.

In recent years, interdisciplinary approaches such as the emerging field of materiobiology have begun to address this gap by designing smart biomaterials capable of modulating both neural and skeletal microenvironments [30]. These include multifunctional scaffolds, nanoengineered surfaces, and bioactive hydrogels that support osteogenesis while delivering neurotrophic or anti-inflammatory factors. Furthermore, the artificial intelligence (AI)-assisted design and modeling of these materials may enable personalized strategies for targeting bone–brain disorders at the systems level.

In this review, we aim to integrate diverse strands of current research to offer a comprehensive overview of the bone–brain axis. We explore how inflammatory signaling, aging, and transcriptional regulation mediate the bidirectional dialog between these systems. We also highlight the therapeutic implications of this interplay, particularly in the context of neurodegenerative diseases and skeletal pathologies, and propose future directions for the rational design of biomaterials and pharmacological agents that can modulate both domains simultaneously. A holistic understanding of this crosstalk holds the promise to reshape current clinical paradigms and foster novel treatment strategies for complex, age-related disorders.

## 2. Similarities Between Bone and Brain Cells

Despite their anatomical and functional differences, bone and brain surprisingly share cellular and molecular homologies, particularly resident cell types that orchestrate tissue remodeling, sensing, and repair. These parallels are not only structural but also reveal shared developmental origins, signaling pathways, and immune-neural functions. Understanding these cross-tissue resemblances may unlock therapeutic opportunities targeting the intersection of skeletal and neurodegenerative disorders.

### 2.1. Osteoclasts and Microglia

Osteoclasts in bone and microglia in the brain both arise from myeloid lineage progenitor cells and function as tissue-resident macrophage-like cells. These cells are specialized in degrading specific types of cells and play a role in remodeling. Whereas osteoclasts resorb bone matrix via acidification and proteases, while microglia prune synapses and clear cellular debris [31]. Intriguingly, both express common markers including cathepsin K, tartrate-resistant acid phosphatase (TRAP), triggering receptor expressed on myeloid cells 2 (TREM2), and receptor activator of nuclear factor kappa-B (RANK)/and its ligand (RANKL) signaling components, revealing shared molecular machinery [32,33]. Mutations in TREM2 or DNAX-activation protein 12 (DAP12) impair both osteoclast activity and microglial function, as observed in Nasu–Hakola disease that presents with bone cysts and early dementia, an archetype of neuroskeletal syndromes [34]. Recent findings suggest that microglia may also engage in bone-like remodeling of the extracellular matrix in pathological conditions such as AD, linking osteoimmune biology to neural degeneration [35].

### 2.2. Osteocytes and Neurons

Osteocytes, embedded within mineralized bone, and neurons, the central players in the nervous system, share profound morphological and functional similarities. Both are long-lived, highly branched, and embedded in a complex cellular network that includes the lacuna-canalicular system in bone and the synaptic network in the brain [36]. At a molecular level, osteocytes and neurons express overlapping sets of genes related to vesicular trafficking, synaptosomal-associated protein 25 (SNAP25) and mitogen-activated protein kinase 1 (SYT1), ion channels, Wnt signaling, and neurotransmitter receptors [37]. Notably, osteocytes secrete sclerostin, a Wnt pathway inhibitor, analogous to neurons for regulating plasticity through synaptic signaling molecules [38]. The presence of gamma-aminobutyric acid (GABA) and glutamate receptors present on osteocytes suggests that bone can directly respond to neurochemical signals. Both cell types also rely on mitochondrial health and oxidative stress regulation for their longevity and function, implicating shared vulnerability pathways in aging [39].

### 2.3. Osteoblasts and Brain Cells

Osteoblasts, responsible for bone matrix synthesis, and certain glial and ependymal cells in the brain share secretory and endocrine roles. Osteoblasts produce OCN, a hormone that crosses the blood–brain barrier and enhances memory, neurotransmitter synthesis, and hippocampal plasticity [5]. Conversely, brain-derived factors like leptin, serotonin, and dopamine regulate osteoblast activity via autonomic nervous system signaling [40]. Developmentally, both osteoblasts and some brain cells are derived from neural crest cells, particularly in craniofacial bones, reinforcing a lineage-level connection [41]. Furthermore, Runx2, a master transcription factor for osteogenesis, also regulates aspects of neurogenesis and memory consolidation, suggesting overlapping regulatory logic [42]. These deep-rooted parallels between bone and brain cells illuminate a functional isomorphism. As transcriptomic and proteomic studies continue to map these similarities, a unifying view of bone and brain as interdependent, co-evolved systems will emerge, shifting our understanding from isolated networks to organ-level synchronicity.

## 3. Signals and Brain Functions

In recent years, a growing body of evidence has converged on the idea that the skeleton functions as an active endocrine organ capable of modulating distant tissues, including the central nervous system (CNS). Among the mediators of this emerging bone-to-brain communication, OCN, osteopontin (OPN), sclerostin, and bone-derived exosomes have emerged as pivotal players. These molecules, through a variety of biochemical and epigenetic mechanisms, contribute to neural development, cognition, mood regulation, and neuroinflammation, thereby integrating into the field of neurobiology.

### 3.1. Osteocalcin

OCN, a non-collagenous metabolic hormone synthesized by osteoblasts, is one of the earliest bone-derived molecules shown to cross the blood–brain barrier and act on the function of the brain. It was reported that OCN not only enhances memory and learning in rodents but also reduces anxiety and supports hippocampal monoamine neurotransmitter synthesis [5]. Mechanistically, these effects are mediated via its interaction with Gpr158, a G-protein-coupled receptor enriched in the hippocampus and brainstem [43], a neuronal receptor for OCN that mediates its effects on cognition and mood regulation [43]. Upon binding of OCN, GPR158 interacts with Gαq, a G protein subunit. This interaction initiates the activation of the inositol 1,4,5-trisphosphate (Gαq-IP3) signaling pathway. While OCN upregulates IP3 expression, it does not affect cyclic AMP (cAMP) expression, indicating a preference for the Gαq-IP3 pathway [44]. Upon binding to GPR158, OCN modulates intracellular signaling pathways that influence monoamine neurotransmitter synthesis and hippocampal-dependent memory formation. In summary, the interaction between GPR158 and OCN involves a complex neuronal signaling pathway that affects BDNF expression, RbAp48 regulation, and synaptic plasticity. This ligand-receptor interaction highlights a direct molecular link between skeletal signaling and central nervous system function, suggesting that changes in bone metabolism can influence brain physiology through defined receptor-mediated pathways [45].

Studies on OCN-deficient mice showed that peripheral OCN can cross the blood–brain barrier (BBB) and bind to multiple brain regions, including the ventral tegmental area, the hippocampal cornus ammonis region 3 (CA3), and the dorsal raphe nucleus [1]. Nevertheless, several factors may influence the interpretation of OCN studies in animal models, including strain-specific knockout effects, such as diet and microbiome, developmental or maternal influences in global knockouts, and variability in biochemical assays detecting different OCN forms [46,47,48]. Recent human studies and Mendelian randomization analyses suggest a possible protective role of OCN in cognition, but the evidence remains inconclusive [49]. Further research using temporally controlled, tissue-specific models, endotoxin-free protein preparations with blood–brain barrier integrity controls, and standardized assays distinguishing OCN forms. Exogenous OCN can activate the synthesis of monoamine neurotransmitters (serotonin, norepinephrine, and dopamine) and brain-derived neurotrophic factor (BDNF) through this pathway, while inhibiting the synthesis of the neurotransmitter GABA, thereby improving cognitive function. Undercarboxylated osteocalcin (uOCN) can cross the BBB and further improve the expression of the BDNF gene and protein in the brain, thereby enhancing cognitive abilities and delaying CNS aging [50,51]. Importantly, circulating levels of osteocalcin decline with age, paralleling age-associated cognitive deterioration [1]. Moreover, maternal OCN during gestation has been shown to influence neurodevelopment in offspring, suggesting that bone-derived signals contribute to brain programming even before birth [5]. This finding not only expands the temporal scope of bone–brain crosstalk but also positions OCN as a critical molecular link between skeletal physiology and cognitive aging.

### 3.2. Osteopontin

OPN, a phosphorylated glycoprotein highly expressed in bone and immune cells, performs a dualistic role in brain physiology. It acts both as a chemokine-like molecule involved in leukocyte migration and as a neuroprotective agent in response to injury. Elevated OPN expression has been observed in various neurodegenerative conditions, including AD, multiple sclerosis, and ischemic stroke, where it is predominantly expressed by activated microglia and astrocytes [52]. OPN promotes the phosphorylation of Akt, accomplishing neuroprotection through the PI3K/Akt pathway in the stroke model [53,54]. OPN also promotes the formation of corpora amylacea in the hippocampus and has a neuroprotective role in AD [55]. OPN-deficient mice showed less severe experimental autoimmune encephalomyelitis (EAE) symptoms. Further studies revealed that this effect results from nuclear OPN-C, produced through thrombin cleavage [53]. In bone, OPN is known to regulate osteoclast adhesion and matrix resorption, but its systemic release can also affect neuroinflammatory circuits. For instance, OPN has been shown to facilitate microglial activation and NF-κB signaling within the CNS, thereby linking skeletal immune activity to neural inflammation [56]. Interestingly, genetic ablation of OPN leads to delayed microglial activation and impaired clearance of amyloid-β plaques, which is associated with AD, indicating that its role in the brain is both context- and time-dependent [57]. The presence of OPN in cerebrospinal fluid (CSF) and its dynamic regulation during disease progression raises the possibility that bone-derived OPN may serve as a biomarker of brain inflammation and as a modifiable target in conditions like traumatic brain injury and neurodegenerative disease [58].

### 3.3. Sclerostin

Sclerostin (SOST), a glycoprotein secreted predominantly by osteocytes, is a potent inhibitor of the canonical Wnt/β-catenin signaling pathway, which is essential for both bone formation and neural plasticity. SOST negatively regulates bone formation by antagonizing the WNT/β-catenin signaling pathway. While SOST itself does not cross the BBB under physiological conditions, its downstream effects are not confined to the skeleton. The Wnt/β-catenin axis is central to adult neurogenesis, oligodendrocyte maturation, and synaptic remodeling; all these processes are compromised during aging and in neurodegenerative diseases [59]. Interestingly, SOST inhibition through monoclonal antibodies such as romosozumab, currently used in osteoporosis therapy, has been shown to enhance cognitive function and protect against stress-induced synaptic loss in a preclinical model [60]. These findings support the idea that systemic manipulation of bone-derived signaling cascades can exert neuroprotective effects, potentially opening new avenues for cognitive enhancement or neuroregeneration. Moreover, recent studies suggest that osteocytes, through sclerostin-mediated paracrine and endocrine communication, can influence systemic metabolic parameters such as glucose homeostasis and adipokine regulation, which are tightly coupled with brain energy metabolism and the risk of neurodegeneration [61].

### 3.4. Exosomes

One of the most versatile and understudied mediators of bone–brain communication is extracellular vesicles (EVs), particularly exosomes derived from osteoblasts, osteoclasts, and bone marrow-derived stromal cells. Exosomes are secreted by different cell types such as lymphocytes, dendritic cells, platelets, mast cells, neurons, macrophages, mesenchymal stem cells (MSCs), and intestinal epithelial cells, significantly contributing to intercellular communication. A common illustration is osteoclasts releasing exosomes that carry RANK, which can attach to its ligand on osteoblasts, thus affecting the process of bone remodeling and healing [1]. Exosomes are capable of crossing the BBB through several mechanisms, such as receptor-mediated transcytosis, macropinocytosis, and adsorptive-mediated transcytosis [62,63]. They also use specific ligand-receptor binding, are internalized into endocytic vesicles, and are then released into the brain parenchyma [62,64]. This ability allows osteoclast-derived exosomes to potentially deliver bioactive molecules to the central nervous system, linking bone physiology with brain function. These nanoscale vesicles carry proteins, lipids, mRNAs, and microRNAs (miRNAs) and modulate gene expression. Bone-derived exosomes have been shown to affect neuronal differentiation, glial activation, and synaptic signaling pathways [65,66]. Emerging studies demonstrate that exosomes enriched in miR-214, miR-29b, or miR-483 can modulate pathways such as PI3K/Akt, BDNF, and MAPK, which are central to neural resilience and plasticity [67]. In particular, osteoblast-derived exosomes carrying miR-214 have been found to inhibit synaptic dysfunction in models of amyloid-induced toxicity, suggesting a protective crosstalk that is amplified under stress or injury [68]. Exosome therapy by itself is an efficient method for addressing neural injuries. Furthermore, advances in the bioengineering of exosomes now allow the targeted delivery of neuroactive cargos, raising the exciting possibility of using bone-derived vesicles as therapeutic shuttles for neurodegenerative diseases. This frontier merges regenerative medicine with inter-organ communication biology, offering a new therapeutic landscape rooted in physiological transport.

## 4. Brain and Bone Interactions

Historically, bone has been regarded as the passive recipient of neural regulation. However, evidence reveals that neurological disorders exert profound and often detrimental effects on skeletal integrity. The brain actively modulates bone remodeling through neuroendocrine, autonomic, and inflammatory axes. Brain-associated pathologies such as traumatic brain injury (TBI), AD, depression, and Nasu–Hakola disease illustrate the interactions of neurocognitive decline or dysregulation with skeletal deterioration, implicating shared molecular mediators and potential bidirectional feedback loops.

### 4.1. Traumatic Brain Injury

TBI has long been paradoxically associated with both heterotopic ossification (HO) and an increased risk of osteoporosis, reflecting a dysregulated neural control over osteogenic pathways. Animal studies show that TBI upregulates bone morphogenetic protein 2 (BMP2) and neuropeptide Y (NPY) in peripheral tissues, stimulating the differentiation of osteoprogenitor cells [69]. Yet chronic or severe injury leads to autonomic dysfunction, muscle atrophy, and impaired mobility, all contributing to osteoporosis [70]. Elevated levels of systemic catecholamines and disrupted hypothalamic–pituitary–adrenal (HPA) signaling following TBI can also reduce bone mass via increased osteoclast activity [70].

### 4.2. Alzheimer’s Disease

AD patients exhibit a higher incidence of osteoporotic fractures, independent of age or activity levels [27]. Intriguingly, amyloid precursor protein (APP) and Aβ peptides are hallmarks of AD, which are also expressed in bone tissue, where they might impair osteoblast differentiation and promote osteoclastogenesis [71]. Additionally, AD-associated cholinergic dysfunction compromises parasympathetic input to the skeleton, tipping the balance toward sympathetic dominance, a known promoter of bone resorption via β2-adrenergic receptors on osteoblasts [3]. These findings underscore a neurodegenerative contribution to skeletal aging.

### 4.3. Depression

Chronic depression is now recognized as an independent risk factor for low bone mineral density. The underlying mechanism is multifactorial: hyperactivation of the HPA axis leads to excess cortisol, which suppresses osteoblastogenesis and accelerates osteocyte apoptosis [72]. Elevated levels of pro-inflammatory cytokines such as IL-6 and TNF-α in major depressive disorder (MDD) further skew bone remodeling toward resorption [73]. Moreover, serotonin dysregulation may directly impact bone via 5-hydroxytryptamine, also known as serotonin (5-HT), receptors on osteoblasts and osteoclasts, suggesting a complex psychoneuroendocrine axis [74].

### 4.4. Nasu–Hakola Disease

A rare but illuminating example of neuro-skeletal crosstalk is Nasu–Hakola disease, caused by mutations in triggering receptors expressed on myeloid cells 2 (TREM2) or tyrosine kinase binding protein (TYROBP), which affect microglial function in the brain and osteoclast differentiation in the bone. Patients exhibit both early-onset dementia and polycystic bone lesions [75]. This genetic overlap highlights the shared immune-osteoclast lineage, positioning microglia and osteoclasts as functionally homologous cells. Findings on TREM2 signaling in Alzheimer’s pathology have opened a new avenue to understanding how innate immune dysfunction might couple neurodegeneration with skeletal degeneration [76].

## 5. Aging and Bone Diseases

The convergence of aging pathways in bone and brain is increasingly evident in clinical settings, where disorders such as osteoporosis and AD often co-occur. These comorbidities are not merely coincidental but reflect a shared vulnerability to chronic inflammation, oxidative stress, hormonal dysregulation, and cellular senescence, which are hallmarks of aging that drive parallel degenerative cascades in both tissues [77,78]. For instance, elderly patients with hip fractures exhibit a significantly increased risk of postoperative cognitive decline and dementia, even in the absence of prior neurodegenerative disease, suggesting systemic crosstalk triggered by trauma and inflammation [79]. Similarly, longitudinal studies demonstrate that individuals with low bone mineral density or a history of osteoporosis have a higher incidence of cognitive impairment, independent of vascular risk factors or physical frailty [80,81]. The physical activity enhances brain resilience through the release of neurotrophic factors such as BDNF, IGF-1, irisin, etc., from muscle and systemic circulation, and the loss of mobility in arthritis patients may diminish these protective signals [82]. Arthritis-brain links are likely the result of both direct skeletal inflammation and secondary systemic and behavioral consequences, with the latter (reduced exercise, effects of medication, and depression) being particularly influential. These patterns suggest bone loss may serve as a prodromal marker for neurodegeneration or reflect underlying systemic pathologies such as chronic low-grade inflammation and mitochondrial dysfunction [83]. The bystander effect, within a biological framework, describes the occurrence where cells not directly affected by a stimulus show biological alterations due to signals received from adjacent cells that were directly impacted [84,85,86]. This effect can be harmful or beneficial, and its consequences are increasingly being examined in relation to different diseases, including those impacting the brain and bones, too. In the context of bone–brain crosstalk, when osteocytes experience mechanical loading or other stimuli, they do not respond in isolation but propagate signals to neighboring cells through their extensive dendritic network. Gap junctions form channels between neighboring cells through the docking of connexons, allowing small ions, including calcium, ATP, and cAMP, to pass through these junctions into adjacent cells, thereby activating signaling pathways in their neighboring cells and forming an osteocytic network through propagate mechanical stimuli [87,88]. The bone–brain bystander communication extends beyond local bone networks to systemic effects. Bone plays a crucial role in maintaining the functionality and balance of various organs by secreting specific cytokines known as osteokines, while these organs reciprocally modulate bone homeostasis and development [89]. This suggests that bystander effects in bone–brain communication can have pathological implications, where dysfunction in one system creates cascading effects in the other through indirect signaling mechanisms. The mechanism highlights the clinical significance of understanding these interconnected bystander responses in both health and disease states.

On a molecular level, bone-derived factors like OCN and lipocalin-2 both decline with age, and have been implicated in neurogenesis, memory, and mood regulation, while elevated sclerostin levels in older adults are associated with cognitive decline and cortical thinking [5,90]. Emerging research also indicates a link between certain brain disorders, such as depression and PD, and increased osteoclastic activity leading to accelerated bone loss. This connection, often referred to as the brain-bone axis, involves complex interactions between the CNS and the skeletal system, partially through hypothalamic–pituitary axis disruption and sympathetic nervous system overactivation [4,91]. Emerging data suggest that systemic interventions targeting senescent cell clearance, gut microbiome modulation, or dual-acting drugs that enhance both neuroplasticity and osteoanabolism may offer novel therapeutic avenues [92,93]. The gut microbiome exerts a dual influence on bone metabolism and neuroinflammation through an emerging gut-bone–brain axis model. Compositional shifts in gut microbiota alter the production of short-chain fatty acids (SCFAs), bile acid derivatives, and tryptophan metabolites, which can modulate osteoclast and osteoblast activity through systemic immune signaling [94]. Perhaps, SCFAs such as butyrate promote regulatory T cell (Treg) expansion in the bone marrow, suppressing osteoclastogenesis through RANKL/OPG modulation [95]. These same immunomodulatory effects extend to the central nervous system, bone marrow-derived myeloid cells, and activated T cells can migrate across the BBB. It influences microglial activation and causes neuroinflammation [96]. Dysbiosis, an imbalance in the gut microbiome, has been linked to both osteoporosis and neurodegenerative disease in human and murine models [97,98]. Experimental evidence shows that antibiotic-induced microbiota depletion reduces bone mass and alters CNS cytokine profiles. At the same time, microbiota restoration or probiotic supplementation reverses such effects [99]. Collectively, these findings support a mechanistic model in which gut microbial metabolites regulate bone marrow immune cell programming, thereby impacting both skeletal integrity and brain inflammation. These approaches aim not only to treat isolated symptoms but also to recalibrate the aging axis that connects bone fragility and brain degeneration, which is a shift toward holistic, age-synchronized medicine.

## 6. Mechanism of Crosstalk

Aging significantly disrupts the communication between bone and brain, contributing to functional decline in both systems. The hypothalamus, which regulates bone homeostasis through the neuroendocrine system, is highly susceptible to aging. Hypothalamic inflammation and senescence affect hormonal signals such as growth hormone, gonadotropins, and sympathetic tone, all of which are regulators of bone remodeling [100,101]. In turn, dysregulated bone-derived signals impact the aging brain. Perhaps OCN declines with age and is known to influence learning, memory, and neurotransmitter synthesis [5,43]. At the same time, this pro-inflammatory environment increases microglial activation and neuronal damage in the brain, impairing cognitive functions [102]. Reduced OCN in the elderly brain may contribute to memory deficits and mood disorders. Other brain-derived signals also show altered activity with aging. For instance, leptin and serotonin, both involved in central regulation of bone mass, show impaired signaling in the aged brain, which may negatively influence bone density [3,4]. Moreover, aging increases oxidative stress and mitochondrial dysfunction in both neurons and osteocytes. These changes lead to cellular senescence and reduced tissue regeneration capacity [92,103]. Furthermore, aging affects the BBB and the bone marrow niche. Disruption of the BBB allows systemic inflammatory cytokines and bone-derived factors to influence brain function, while bone marrow aging reduces the output of hematopoietic and mesenchymal stem cells, impairing bone repair and immune regulation [104,105]. These aging-related impairments create a feedback loop in that dysfunction in bone accelerates brain aging, and vice versa. In summary, aging weakens the bone–brain crosstalk through hormonal, inflammatory, and oxidative pathways. Understanding these mechanisms is essential for developing interventions that target age-related osteoporosis and neurodegeneration together.

## 7. Brain Mediators and Cellular Functions

The skeletal system is densely innervated by sensory and autonomic nerve fibers, allowing the brain to influence bone function through a variety of neurotransmitters and neuropeptides. These mediators not only regulate pain and movement but also directly impact bone cell activity. Norepinephrine, released from sympathetic nerves, binds to β2-adrenergic receptors on osteoblasts, reducing bone formation and enhancing resorption, a mechanism heightened under chronic stress or neurodegenerative disease [106]. Similarly, serotonin, synthesized both peripherally and centrally, plays a dual role; brain-derived serotonin promotes bone mass, while gut-derived serotonin inhibits osteoblast function [107]. Recent studies have identified calcitonin gene-related peptide (CGRP) and substance P, released from sensory nerves, as positive regulators of osteogenesis. These peptides stimulate osteoblast proliferation and inhibit osteoclast formation, pointing to a protective role in maintaining bone integrity under mechanical or inflammatory stress [108,109]. One emerging area is the influence of BDNF, a molecule best known for synaptic plasticity, which has now been shown to enhance osteoblast differentiation and bone healing. BDNF may also act through tropomyosin receptor kinase B (TrkB) receptors expressed on bone marrow stem cells, linking neuronal activity to skeletal regeneration [110,111,112]. Furthermore, recent evidence suggests that exosomal transfer of miRNAs and proteins from neurons to bone cells offers a novel mechanism for long-distance brain-bone communication that bypasses traditional signaling routes [66,113,114]. These findings suggest that neurochemical signals from the brain and peripheral nerves are not merely modulators of pain or movement but active participants in skeletal remodeling, especially during injury, aging, and disease.

## 8. Stem Cells and Crosstalk

Recent studies suggest that stem cells are not only central to tissue repair but also act as mediators in the communication between the brain and bone. MSCs from bone marrow, long known for forming bone, also respond to neural signals such as serotonin, dopamine, and BDNF, which influence their ability to become bone cells [115]. This shows that mental and emotional states can indirectly affect bone remodeling by controlling the behavior of stem cells. In return, bone-derived MSCs release exosomes, like small vesicles that carry proteins, mRNAs, and miRNAs, which can reach the brain and modulate processes like neurogenesis and synaptic plasticity [116]. For instance, exosomal miR-29b and miR-146a have been shown to reduce neuroinflammation in models of AD and also promote osteogenic differentiation [117]. Interestingly, neural stem cells (NSCs) in the brain also share similar signaling pathways with bone-forming MSCs, especially in aging and stress conditions. Pathways such as Wnt, Notch, and Sonic Hedgehog (Shh) operate in both niches and are influenced by systemic inflammation and hormonal changes [118]. Neurotrophic factors such as BDNF, nerve growth factor (NGF), and vascular endothelial growth factor (VEGF) secreted by MSCs play roles in both brain plasticity and bone vascularization, bridging the gap between the two organ systems. Moreover, in conditions like TBI, circulating MSCs migrate to both the brain and bone injury sites, suggesting a shared repair mechanism between the two tissues [119]. This dual targeting opens the possibility of using stem cells or engineered stem cells for therapies that will address both neurodegeneration and bone loss. Together, these findings highlight stem cells as key players in bone–brain crosstalk, not just as repair agents, but as interpreters of biochemical messages shared between the two systems.

## 9. Extracellular Vesicles and Brain Cell Function

EVs, including exosomes and microvesicles, have emerged as key mediators in the communication between bone and the brain. These nanoscale vesicles, secreted by various cell types such as osteoblasts, osteocytes, neurons, and glia, carry bioactive cargo such as proteins, lipids, mRNAs, and non-coding RNAs that influence distant cellular behavior [120]. Findings show that bone-derived EVs can cross the BBB and modulate neural activity. For instance, osteocyte-derived exosomes enriched with miR-129-5p have been shown to reduce neuroinflammation by targeting pro-inflammatory genes in microglia [15]. Conversely, brain-derived EVs released during neurodegenerative diseases, like Alzheimer’s, carry inflammatory mediators that impair osteoblast function and accelerate bone loss. Interestingly, EVs from MSCs or induced pluripotent stem cells (iPSCs) derived osteoblasts promote both bone regeneration and neuronal protection in aging models [121]. These dual effects are mainly due to EV-contained factors like miR-21, miR-146a, and TGF-β1, which regulate shared pathways such as NF-κB, Wnt/β-catenin, and PI3K-AKT signaling in both tissues. Although genes associated with vesicle trafficking and mitochondrial function are typically expressed in metabolically active cells, osteocytes seem to have an especially significant dependence on these processes for their particular roles in bone maintenance and remodeling [122]. Emerging evidence suggests that EV cargo reflects the aging status of bone and brain tissues. Aged osteocyte-derived EVs carry elevated senescence-associated miRNAs (miR-34a, miR-483), which impair synaptic plasticity and reduce osteogenesis, thereby contributing to both cognitive decline and skeletal fragility [123]. These findings propose EVs as not only biomarkers of bone and brain health but also as targets for therapeutic intervention. Moreover, engineering EVs to deliver specific cargo such as siRNAs, CRISPR/Cas9 components, or neurotrophic factors is an evolving strategy in regenerative medicine that could bridge neurodegeneration and osteoporosis treatment [124]. These vesicles offer a nonimmunogenic, stable, and targetable means to modulate the bone–brain axis in age-related and inflammatory diseases.

## 10. Metabolic Disorders and Crosstalk

Metabolic disorders such as obesity, type 2 diabetes mellitus (T2DM), and metabolic syndrome disrupt the bidirectional communication between bone and brain. These disorders are often associated with chronic systemic inflammation, which impairs both bone remodeling and neuronal health [125]. In obesity and T2DM, increased levels of pro-inflammatory cytokines like TNF-α and IL-6 lead to enhanced bone resorption and inhibit osteoblast differentiation, contributing to osteoporosis. Simultaneously, these cytokines cross the blood–brain barrier and promote neuroinflammation, oxidative stress, and cognitive impairment [126]. Insulin resistance, a hallmark of T2DM, alters osteoblast function and decreases bone formation. In the brain, insulin resistance contributes to impaired synaptic signaling, memory deficits, and has been linked to AD pathology. Moreover, high levels of circulating glucose and advanced glycation end products (AGEs) reduce bone strength and promote neuronal damage. Bone-derived hormones like OCN are reduced in metabolic disorder mice, which further impairs cognitive functions such as learning and memory, whereas OCN enhances neurotransmitter synthesis in the brain [127,128]. On the other hand, brain-derived hormones, including leptin and serotonin, which regulate bone metabolism via central pathways, show reduced and promoted expression in obesity and T2DM, respectively. Leptin resistance in the hypothalamus impairs bone formation and disrupts energy homeostasis. Additionally, metabolic disorders accelerate cellular senescence and mitochondrial dysfunction in both bone and brain tissues. The bone marrow microenvironment is also altered in metabolic syndrome, leading to impaired hematopoiesis and inflammatory factors in the cells, which further affects the central nervous system [3,107,129,130]. Thus, metabolic dysregulation establishes a pathological link between bone fragility and brain dysfunction, emphasizing the need for integrated therapeutic strategies.

## 11. Osteoimmunology and Crosstalk

Osteoimmunology, an interdisciplinary field coined to describe the cross-regulation between bone cells and the immune system, encompasses the shared mechanisms and interactions between bone cells and immune cells, with RANKL serving as an osteoclast differentiation factor that directly links activated immune responses to bone loss [131]. The fundamental basis of osteoimmunology emerges from the recognition that bone-resorbing osteoclasts belong to the monocyte/macrophage lineage and that RANKL functions as an essential regulator for both immune organ development and osteoclast differentiation, representing the cytokine that forms the foundation for osteoimmunology following its discovery [31]. This molecular convergence has established osteoimmunology as an interdisciplinary research field focused on understanding the interplay between skeletal and immune systems, where a large number of molecules affect both bone and immune cells through mechanisms mediated not only by cytokine and chemokine release but also through direct cell–cell contact [132].

Tissue-resident macrophages, including osteoclasts and microglia, represent highly specialized cells adapted to their respective tissue-specific microenvironments. These macrophages, activated by various inflammatory signals, are responsible for pathological changes in osteoporosis and AD in bone and brain conditions that demonstrate increased co-occurrence compared to the general population prevalence [133]. The shared developmental origins and functional characteristics of these myeloid-derived cells establish a mechanistic foundation for osteoimmune regulation of bone–brain communication through common signaling pathways and inflammatory responses. Apart from this, the osteoimmune system demonstrates complex integration with neuroinflammatory processes, exemplified by Th17 TNF-α+ cells that migrate to bone marrow and promote osteoclast progenitor recruitment during inflammatory bowel disease, with T cells mediating crosstalk between inflamed intestinal tissues and bone destruction through paralleled upregulation of TNF-α and RANKL in both gut and bone compartments [134].

**Figure 1:**
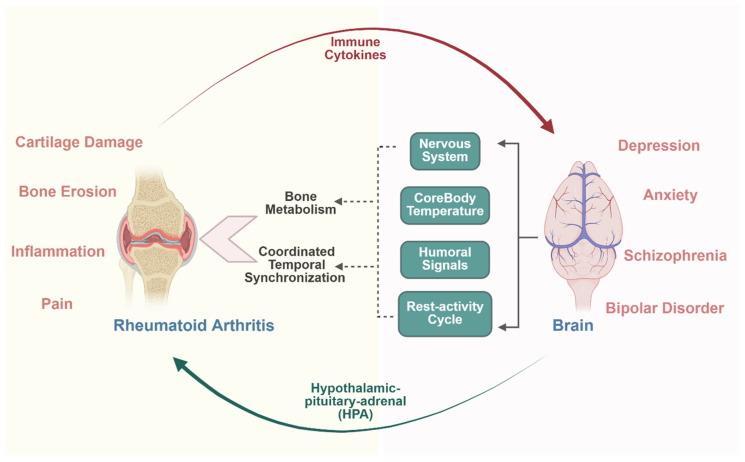
Bone–brain crosstalk in rheumatoid arthritis: a bidirectional communication network. This schematic illustrates the complex bidirectional communication pathways between bone and brain systems in rheumatoid arthritis. The bone compartment (**left**) shows characteristic pathological features, including cartilage damage, bone erosion, inflammation, and pain associated with rheumatoid arthritis. The brain compartment (**right**) displays various neuropsychiatric manifestations commonly observed in RA patients, including depression, anxiety, schizophrenia, and bipolar disorder. Central mediating factors include bone metabolism regulation, coordinated temporal synchronization, and the rest-activity cycle, which facilitate communication through multiple pathways: the nervous system (autonomic and sensory pathways), core body temperature regulation, and humoral signaling (hormones and cytokines). The hypothalamic–pituitary–adrenal (HPA) axis serves as a major neuroendocrine pathway mediating stress responses and inflammatory processes. Immune cytokines released from inflamed joints create a systemic inflammatory environment that affects brain function and mood regulation.

Rheumatoid arthritis exemplifies pathological osteoimmune dysregulation, where ongoing inflammatory conditions disrupt the equilibrium between osteoclasts and osteoblasts through immune system hyperactivation, leading to excessive osteoclast activity that results in localized bone erosion, periarticular bone weakness, and systemic osteoporosis, as shown in Figure 1. Contemporary osteoimmunological research has expanded to encompass the crosstalk between bone and immune cells in both physiological and pathological conditions, with mechanisms grouped into categories including general regulatory processes, disease-specific interactions, and therapeutic interventions [135]. Understanding osteoimmunological mechanisms in bone–brain crosstalk has revealed promising therapeutic strategies, including enhancing microglial phagocytosis, reducing microglial-mediated neuroinflammation, inhibiting microglial exosome synthesis and secretion, and promoting microglial conversion into protective phenotypes for neurodegenerative disease treatment [136,137].

## 12. Inflammation and Brain Cells

Inflammation is a central and bidirectional mediator in the bone–brain axis, linking neurodegenerative disorders and skeletal pathologies through shared molecular pathways. Chronic, low-grade inflammation is often referred to as inflammaging, which disrupts both bone homeostasis and cognitive function in aging and disease. Some signals are proven endocrine messengers in vivo, such as osteocalcin, lipocalin-2, sclerostin, leptin-sympathetic circuits, and CGRP, while those still with supportive in vitro or correlative data, such as cytokine spillover (IL-6, TNF-α, IL-1β) and bone-derived exosomes. Studies show that pro-inflammatory cytokines, including IL-1β, TNF-α, and IL-6, not only contribute to osteoclast activation and bone loss but also promote microglial activation and neurodegeneration. For example, elevated IL-6 levels are associated with both hip fracture risk and cognitive decline, indicating a common inflammatory signature in both organs [77]. Importantly, bone cells themselves, particularly osteocytes and osteoclasts, release inflammatory mediators that influence the brain. Osteoclast-derived high mobility group box 1 (HMGB1), a damage-associated molecular pattern (DAMP), has been shown to activate toll-like receptor (TLR) 4 signaling in microglia and impair memory formation in a mouse model. Conversely, neuroinflammation in AD can elevate RANKL expression, enhancing osteoclastogenesis and increasing fracture risk. Emerging data also highlight the aging of senescence-associated secretory phenotype (SASP) factors in both tissues. Senescent bone and brain cells release similar pro-inflammatory signals, such as IL-8 and monocyte chemoattractant protein (MCP) 1, that propagate tissue dysfunction and systemic inflammation [27]. Furthermore, nucleotide-binding domain, leucine-rich containing family, pyrin domain containing-3 (NLRP3) inflammasome activation has been identified as a shared mechanism driving both bone resorption and neuronal damage in aging and metabolic diseases. New insights from single-cell transcriptomics suggest that inflammatory genes co-regulate both osteoimmune and neuroimmune responses, linking NF-κB, STAT3, and IRF signaling in both cell types [138]. These findings support a model where inflammation acts not just as a byproduct, but as a regulatory bridge between bone and brain health.

## 13. Transcriptional Regulators and Brain Cells

At the heart of bone–brain communication lies a shared network of molecular mediators and transcriptional regulators that coordinate cellular responses across both systems. Recent studies show that bone and brain cells express overlapping signaling pathways involved in stress response, differentiation, and inflammation, which may explain their interconnected aging and disease processes [139,140]. One key mediator is NF-κB, a transcription factor activated by inflammation. In both osteoclasts and microglia, NF-κB drives inflammatory gene expression, contributing to bone resorption and neurodegeneration, respectively. Similarly, FOXO3, known for its role in oxidative stress resistance, regulates bone homeostasis and supports neuronal survival under aging and metabolic stress. SIRT1, NAD-dependent deacetylase, plays a dual role: it suppresses osteoclastogenesis while promoting synaptic plasticity and neuroprotection. Loss of SIRT1 with age is associated with both osteoporosis and cognitive decline [141,142]. Additionally, cyclic AMP response element-binding protein (CREB) is crucial in memory formation and is also involved in osteoblast differentiation, linking learning-related gene expression with bone formation processes. Transcriptomics has revealed that both neurons and osteocytes express genes related to vesicle trafficking, Wnt signaling, and mitochondrial function, highlighting the shared energy demands and communication mechanisms between these two cell types. Notably, Wnt/β-catenin signaling, essential for neurogenesis, is equally critical for bone formation. Disruption of this pathway is implicated in both AD and osteopenia, suggesting that systemic Wnt modulators may serve dual therapeutic roles [37,143,144]. These molecular overlaps support the idea that bone and brain are not just physically linked, but molecularly synchronized, particularly during stress, aging, and disease. Understanding these shared transcriptional regulators opens new paths for treating disorders that affect both systems simultaneously [145] (Table 1).

## 14. KLFs and Crosstalk

Kruppel-like factors (KLFs) are transcription factors that control many biological processes such as cell growth, inflammation, and differentiation [146,147,148,149,150,151,152]. In recent years, KLFs, particularly KLF2, KLF4, and KLF5, have been found to influence both bone and brain health, pointing to a shared regulatory network. In the skeletal system, KLF2 and KLF4 promote osteoblast differentiation and reduce inflammation by downregulating NF-κB and RANKL signaling, which suppresses bone resorption [152,153]. At the same time, in the brain, KLF2 maintains neuronal integrity and protects against inflammatory insults by modulating cytokine expression and microglial activation [154]. The mechanotransduction and metabolic control make KLF2 particularly interesting in bone–brain communication. Mechanical loading upregulates KLF2 in both endothelial and bone cells, suggesting that physical stimuli may coordinate bone and brain responses to KLF2-mediated pathways [155]. Furthermore, KLF2 enhances mitochondrial function and reduces oxidative stress, both key mechanisms of image-related decline of skeletal and neural tissues [151,156,157,158,159]. Additionally, KLF2 expression is influenced by circulating microRNAs and exosomal signaling, hinting at a systemic mode of regulation that could facilitate inter-organ communication [148].

KLFs also act in the brain to maintain blood–brain barrier integrity, reduce oxidative damage, and protect neurons from inflammatory stress [160,161]. Interestingly, KLF4 is also expressed in neural stem cells and has been linked to synaptic plasticity and learning. In both neurons and osteoblasts, KLF4 appears to enhance mitochondrial biogenesis and reduce ROS, showing a conserved stress response in these two tissue types. Moreover, KLF5, although better known for its role in cancer biology, has been shown to regulate mesenchymal stem cell differentiation toward osteogenic or neural fates depending on extracellular signals [162]. This suggests that KLFs may integrate environmental cues to coordinate developmental pathways in bone and brain. Emerging data also suggest that KLF expression is modulated by exercise, diet, and mechanical loading, which are known to benefit both skeletal and cognitive function. Thus, KLFs could act as key molecular links that translate systemic signals into coordinated responses in bone and brain [163,164].

## 15. Biomarkers and Crosstalk

**Figure 2:**
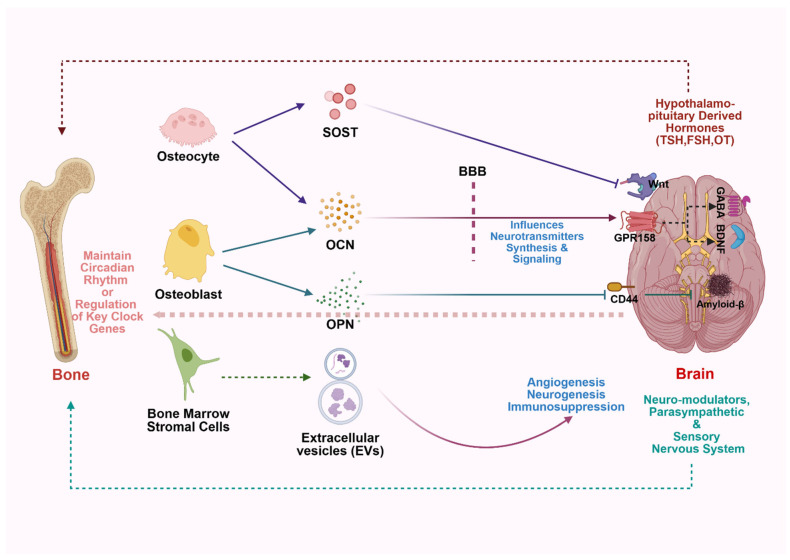
Mechanisms and potential modulation of bone–brain interaction: a comprehensive overview of cellular and molecular pathways. The bone compartment (**left**) contains key cellular players, including osteocytes, osteoblasts, and bone marrow stromal cells, which collectively maintain circadian rhythm regulation of key clock genes. Osteocytes release sclerostin (SOST), while osteoblasts secrete osteocalcin (OCN) and osteopontin (OPN), serving as bone-derived signaling molecules. Bone marrow stromal cells contribute through extracellular vesicles (EVs) that facilitate intercellular communication. The brain compartment (**right**) shows the blood–brain barrier (BBB) as a critical interface, with hypothalamo-pituitary-derived hormones providing endocrine regulation. Key neuronal markers include Wnt signaling pathways, GPR158- G protein-coupled receptor 158, GIRK channels, CD44 surface receptors, and amyloid-β deposits, highlighting the molecular complexity of neural responses to bone-derived signals. Note: BDNF—brain-derived neurotrophic factor; GABA–gamma-aminobutyric acid.

Understanding the molecular links between bone and the brain has revealed new opportunities for therapeutic targeting and biomarker discovery, especially in age-related diseases such as osteoporosis and neurodegeneration. OCN has emerged not only as a biomarker of bone turnover but also as a modulator of cognition and anxiety. Clinical studies suggest that serum OCN levels correlate with hippocampal activity and memory performance in older adults [5,165]. Similarly, sclerostin, traditionally known to inhibit bone formation, is now being explored for its effects on cerebral perfusion and synaptic plasticity. Anti-sclerostin antibodies (romosozumab) used in osteoporosis treatment may also impact cognitive function, although this requires further investigation [166]. As previously discussed, exosomal microRNAs are emerging as dual-system biomarkers, found in both bone and brain pathology. Small molecules like miR-34a and miR-124 have been linked to osteoblast inhibition and neuroinflammation, making them potential diagnostic tools and therapeutic targets in diseases like Alzheimer’s and osteoporosis [167]. These exosomes could also serve as delivery vehicles for targeted therapies that modulate both skeletal and neural microenvironments. Another promising biomarker is the NLRP3 inflammasome, a key inflammatory node active in both osteoclasts and microglia. Trials on NLRP3 inhibitors, such as MCC950, show potential in reducing both bone loss and neurodegeneration, positioning it as a shared therapeutic target. AI-driven biomarker discovery has further accelerated progress in this area. Multi-omics studies integrating proteomics, epigenomics, and single-cell RNA-seq data are identifying tissue-shared gene signatures, including KLF2, FOXO3, and NF-κB, that are disrupted in both systems, as shown in Figure 2 [168]. Overall, these insights offer hope for cross-beneficial therapies and predictive biomarkers that bridge the current gap between neurology and orthopedics. Targeting shared pathways may lead to dual-action drugs that restore both skeletal integrity and cognitive health.

## 16. Signaling Pathways

Previously, scientists considered bone as a passive structure. Now we know the bone is an active endocrine organ. This discovery has changed the understanding of skeletal biology. Bone actively controls body metabolism through OCN. Advanced molecular omics technologies help scientists study communication pathways. These pathways allow the brain and bone to communicate with each other [169,170]. Investigations have elucidated the mechanistic basis of OCN action, demonstrating its capacity to cross the BBB and directly influence hippocampal neurons. Upon binding to Ca^2+^, OCN functions as a mineralization inducer by promoting the deposition of phosphate PO43^−^, ultimately facilitating hydroxyapatite formation, while simultaneously serving as a circulating hormone that modulates brain function. OCN affects the brain through specific receptors in the nervous system. Recent studies showed that the OCN improves brain functions. It helps to create new brain cells in the hippocampus. It also strengthens connections between brain cells and improves memory through activation of cAMP-dependent signaling cascades. These findings establish OCN as a bona fide bone–brain axis hormone with direct implications for cognitive health and neurodegeneration [45,50].

The leptin-sympathetic system is a key pathway. It showed how the brain controls bone metabolism and showed that leptin reduces bone formation. It also works through central nervous system pathways, not by directly affecting bone cells to activate sympathetic nerves. Sympathetic signaling via beta2-adrenergic receptors (Adrb2) present on osteoblasts controls bone formation downstream of leptin. This mechanism demonstrates how metabolic hormones from adipose tissue can influence skeletal homeostasis through neural intermediates. It creates a three-way communication between adipose tissue, brain, and bone. Further complexity emerges from research demonstrating that without leptin, sympathetic activity decreases [4,171].

Beyond the leptin-sympathetic axis, multiple neurotransmitter systems contribute to bone–brain crosstalk. The cocaine and amphetamine-regulated transcript (CART) represents one such molecular mediator that links central nervous system activity to peripheral bone metabolism. The sympathetic nervous system favors bone resorption by increasing the expression in osteoblast progenitor cells of CART and related signaling molecules [171]. Serotonin pathways are important for bone–brain communication. Serotonin in the brain and serotonin in the gut both affect bone health. They work through different mechanisms. Brain serotonin affects bone formation by controlling sympathetic nerves. Gut serotonin directly affects bone cells through specific receptors. This dual-pathway system explains why mood disorders and antidepressant drugs affect bone density. Studies indicate a complex relationship between depression, antidepressants, and fracture. First, the presence of depression itself increases fracture risk, in relation with decreased bone mineral density and an increase in falls, while the pharmacological intervention through selective serotonin reuptake inhibitors affects both central serotonergic pathways (influencing sympathetic outflow to bone) and peripheral serotonin systems (directly acting on bone cell receptors), explaining why both the underlying mood disorder and its treatment independently contribute to bone density changes [172,173].

The bone morphogenetic proteins (BMPs) constitute a group of potent morphogens that are critical for the patterning, development, and function of the central nervous system. While the Wnt and BMP pathways are evolutionarily conserved and essentially independent signaling mechanisms that often regulate similar biological processes. TGF-β/BMPs work by activating receptor serine/threonine kinases and are important for bone health. When these proteins do not work properly, developmental problems occur. TGF-β/BMP signaling controls many cell processes and is fundamentally important during the entire life of all metazoans. These proteins can both build and break down bone and cartilage. They also control the behavior of bone-forming cells. This system works through Smad proteins. TGF-β activates Smad2/3 proteins, whereas BMP activates Smad1/5/8 proteins. These signals combine to coordinate bone–brain communication. Wnt and BMP signaling work together in many processes, including embryo development. These pathways form a turbulent relationship characterized by complex crosstalk mechanisms. Notch and Wnt signals work together during bone healing. They control how bone precursor cells grow and develop, which demonstrates the interconnected nature of these signaling networks in bone formation. The main Wnt pathway uses the β-catenin protein. This protein moves to the cell nucleus and turns on bone-building genes. The same pathway also affects brain development through shared mechanisms [174,175,176]. Also, direct vascular channels connect the skull marrow to the meninges, enabling rapid, local myeloid trafficking to the brain surface that can bypass systemic circulation. An anatomy not described for the gut or peripheral immune organs, supplying the meninges to the same extent [177].

Recent transcriptome approaches have demonstrated TREM2/ DNAX-activating protein of 12 kDa (DAP12) signaling as one of the principal regulators that transform microglia from a homeostatic to a neural disease-associated state. TREM2 and DAP12 form an immunoreceptor signaling complex. This complex regulates the development, activation, and function of myeloid cells, including microglia and osteoclasts. In osteoclasts, TREM2-DAP12 signaling controls multinucleation, migration, and bone resorption. In microglia, it contributes to activation and functional responses. The DAP12 is a signaling adapter protein expressed in cells that participate in innate immune responses, pairing with different receptors to regulate the JNK signaling pathway. This dual functionality establishes TREM2-DAP12 as a critical molecular bridge linking bone remodeling and neuroinflammatory processes [178,179]. Furthermore, the TGF-β and Notch signaling pathways play a key role in fibrogenesis, identifying molecular mechanisms that reveal numerous factors and signaling pathways involved, though the interactions between these pathways remain unclear [180]. Notch signaling, through its canonical pathway involving γ-secretase-mediated cleavage and nuclear translocation of the Notch intracellular domain (NICD), regulates both osteoblast differentiation and neural stem cell fate determination [181,182].

## 17. Pre-Clinical and Clinical Studies

Preclinical studies have shown that bone-derived factors directly affect brain function. OCN crosses the BBB and enhances memory, learning, and mood regulation in mouse models. In OCN-deficient mice, increased anxiety and impaired memory were observed, suggesting its neuroprotective role [5]. Experimental infusion of OCN in aged or cognitively impaired mice reversed these deficits, highlighting therapeutic potential. FGF23, another bone-derived hormone, affects hippocampal function and has been associated with cognitive decline in mice with chronic kidney disease, which is a condition involving altered bone metabolism. Preclinical studies also showed that RANKL inhibitors (denosumab) may reduce neuroinflammation, suggesting dual action on bone and brain [45,183]. In the reverse direction, brain-derived signals also influence bone remodeling. In mouse models, hypothalamic damage or inflammation impairs bone formation through disrupted hormonal signaling and increased sympathetic tone. Central administration of leptin was shown to reduce bone mass through sympathetic nervous system activation. Patients with neurodegenerative diseases show reduced bone mineral density. They also have increased fracture risk, particularly in AD and PD [3,184,185]. A meta-analysis reported ~2.5-fold greater odds for bone fracture risk, and nationwide cohorts show adjusted risk ratios around 1.8 [186,187,188]. A clinical study examined elderly patients. Those with lower bone mineral density had worse cognitive performance. They also showed more brain changes on MRI scans. Osteoporosis patients develop dementia more often. Similarly, dementia patients develop osteoporosis more often. This suggests both diseases share common causes. Bone medications like bisphosphonates and denosumab may reduce dementia risk. Observational studies support this finding. A meta-analysis of 26 cohorts found that people with a prior fracture had a higher subsequent dementia risk [189]. Dementia also worsens post-fracture outcomes, with ~67% higher 30-day postoperative mortality after hip-fracture surgery compared to non-dementia peers [190]. Although anti-resorptive drugs such as bisphosphonates are widely used to prevent fractures in osteoporosis, some studies have suggested potential negative effects on neuroprotection. Long-term bisphosphonate use may alter mevalonate pathway signaling in the brain, influence microglial activation, or impair synaptic remodeling, potentially counteracting neuroprotective processes [87,191]. These observations remain inconclusive but highlight the need for careful evaluation of bone-targeted therapies in populations at risk for dementia. However, we cannot prove these drugs directly prevent dementia. Long-term steroid use damages both brain function and bone health. This provides strong evidence for bone–brain connections [192,193,194]. Collectively, these findings from preclinical and clinical studies support the existence of bone–brain crosstalk and its relevance to aging and chronic diseases. They also suggest that bone-targeted therapies may offer cognitive benefits, and vice versa.

## 18. Conclusions

The bone and brain communicate through a complex network of hormones, cytokines, and neural signals. Bone-derived signals influence brain development, cognition, and behavior, while brain and nerve-derived mediators regulate bone remodeling. Inflammation, aging, and metabolic changes disrupt this crosstalk, leading to both skeletal and neurological decline. OCN, FGF23, and lipocalin-2 are key bone-derived factors influencing brain function, while brain-derived signals like leptin and serotonin regulate bone remodeling. Aging and chronic inflammation enhance oxidative stress and impair both systems. Clinical and preclinical evidence support this bidirectional relationship. Stem cells, extracellular vesicles, and shared molecular pathways play central roles in this communication. Recognizing common biomarkers and regulatory mechanisms offers new insights into age-related disorders. This review is important as it highlights the emerging link between skeletal and neural health, offering novel insights into shared mechanisms that can guide future research and therapeutic strategies for aging and chronic diseases. Understanding this crosstalk may help in early diagnosis and dual-targeted interventions for both bone and brain disorders.

## 19. Limitations and Future Directions

Despite progress in bone–brain crosstalk research, it still faces several limitations. Most studies are based on animal models, which may not fully reflect human physiology. The specific receptors and transport mechanisms for bone-derived hormones in the brain remain incompletely defined. The signaling pathway of OCN in different brain regions is still unclear. There is also a limited understanding of how aging, sex hormones, and metabolic disorders alter this communication over time. Another challenge is the lack of longitudinal human studies that link bone biomarkers to brain structure or function. Current clinical data are mostly observational, making it difficult to determine causality. Moreover, the role of neurodegenerative disease in altering bone metabolism is still not fully explored.

In future studies, combining multi-omics, neuroimaging, and bone phenotyping could help identify the in-depth mechanisms. Human trials assessing the cognitive effectiveness of targeted drugs like bisphosphonates or osteocalcin analogs may offer therapeutic insights. Understanding sex-specific and age-specific variations in bone–brain signaling will also be essential. Finally, expanding research into immune and vascular mediators could uncover new links in the bone–brain axis.

## Figures and Tables

**Table 1:** Summary of molecular mediators of bone–brain crosstalk. Note: GH—growth hormone, TSH—thyroid-stimulating hormone, FSH—follicle-stimulating hormone, AVP—arginine-vasopressin, PRL—lactotrophic cell-derived prolactin, AgRP—neuropeptide agouti-related peptide, CART—cocaine amphetamine-regulated transcript, POMC—proopiomelanocortin, VIP—vasoactive intestinal peptide, NPY—neuropeptide y, Ach—acetylcholine, DA—dopamine, GLU—glutamate, LCN2—lipocalin 2, DKK1—Dickkopf-related protein 1, IGF-1—insulin-like growth factor 1.

Predominant Mediator Effect	Types of Secretory Factors	Regulatory Mediators
**Brain-derived**	Neurohormones	GH, Melatonin, TSH, OH, FSH, AVP, PRL
	Neuropeptides	AgRP, CART, POMC, VIP, NPY
	Neurotransmitters	Ach, DA, GLU, Serotonin
	Sensory Innervation	CGPR, Semaphorin 3A
**Bone-derived**	Hormones	OCN, LCN2
	Peptide	Sclerostin
**Adipocyte-derived**	Hormones	Adiponectin, Leptin
**Locally Synthesized Mediators**	Peptide	DKK1, OPN, RANKL, Irisin
	Growth Factors	BDNF, IGF-1, BMPs

## Data Availability

No new data were created or analyzed in this study.

## References

[1] Karsenty G., Ferron M. (2012). The contribution of bone to whole-organism physiology. Nature.

[2] Lee N.K., Sowa H., Hinoi E., Ferron M., Ahn J.D., Confavreux C., Dacquin R., Mee P.J., McKee M.D., Jung D.Y. (2007). Endocrine regulation of energy metabolism by the skeleton. Cell.

[3] Takeda S., Elefteriou F., Levasseur R., Liu X., Zhao L., Parker K.L., Armstrong D., Ducy P., Karsenty G. (2002). Leptin regulates bone formation via the sympathetic nervous system. Cell.

[4] Ducy P., Amling M., Takeda S., Priemel M., Schilling A.F., Beil F.T., Shen J., Vinson C., Rueger J.M., Karsenty G. (2000). Leptin inhibits bone formation through a hypothalamic relay: A central control of bone mass. Cell.

[5] Oury F., Khrimian L., Denny C.A., Gardin A., Chamouni A., Goeden N., Huang Y.-y., Lee H., Srinivas P., Gao X.-B. (2013). Maternal and offspring pools of osteocalcin influence brain development and functions. Cell.

[6] Oury F., Sumara G., Sumara O., Ferron M., Chang H., Smith C.E., Hermo L., Suarez S., Roth B.L., Ducy P. (2011). Endocrine regulation of male fertility by the skeleton. Cell.

[7] Schett G. (2011). Effects of inflammatory and anti-inflammatory cytokines on the bone. Eur. J. Clin. Investig..

[8] Perry V.H., Cunningham C., Holmes C. (2007). Systemic infections and inflammation affect chronic neurodegeneration. Nat. Rev. Immunol..

[9] Kinney J.W., Bemiller S.M., Murtishaw A.S., Leisgang A.M., Salazar A.M., Lamb B.T. (2018). Inflammation as a central mechanism in Alzheimer’s disease. Alzheimer’s Dement. Transl. Res. Clin. Interv..

[10] Hansda S., Prateeksha P., Das H. (2024). Krüppel-like factor 2 (KLF2), a potential target for neuroregeneration. Neural Regen. Res..

[11] Franceschi C., Campisi J. (2014). Chronic inflammation (inflammaging) and its potential contribution to age-associated diseases. J. Gerontol. Ser. A Biomed. Sci. Med. Sci..

[12] Almeida M. (2012). Aging mechanisms in bone. BoneKEy Rep..

[13] Li X.-h., Chen C., Tu Y., Sun H.-t., Zhao M.-l., Cheng S.-x., Qu Y., Zhang S. (2013). Sirt1 promotes axonogenesis by deacetylation of Akt and inactivation of GSK3. Mol. Neurobiol..

[14] Salminen A., Huuskonen J., Ojala J., Kauppinen A., Kaarniranta K., Suuronen T. (2008). Activation of innate immunity system during aging: NF-kB signaling is the molecular culprit of inflamm-aging. Ageing Res. Rev..

[15] Wang X., Wei Z., Jiang Y., Meng Z., Lu M. (2021). mTOR signaling: The interface linking cellular metabolism and hepatitis B virus replication. Virol. Sin..

[16] Schweingruber C., Nijssen J., Mechtersheimer J., Reber S., Lebœuf M., O’Brien N.L., Mei I., Hedges E., Keuper M., Benitez J.A. (2025). Single-cell RNA-sequencing reveals early mitochondrial dysfunction unique to motor neurons shared across FUS- and TARDBP-ALS. Nat. Commun..

[17] Wang J.S., Kamath T., Mazur C.M., Mirzamohammadi F., Rotter D., Hojo H., Castro C.D., Tokavanich N., Patel R., Govea N. (2021). Control of osteocyte dendrite formation by Sp7 and its target gene osteocrin. Nat. Commun..

[18] Pulimood N.S., Rodrigues W.D.S.J., Atkinson D.A., Mooney S.M., Medina A.E. (2017). The Role of CREB, SRF, and MEF2 in Activity-Dependent Neuronal Plasticity in the Visual Cortex. J. Neurosci..

[19] Tang K.C., Pan W., Doschak M.R., Alexander R.T. (2019). Increased FoxO3a expression prevents osteoblast differentiation and matrix calcification. Bone Rep..

[20] Liu T.M., Lee E.H. (2013). Transcriptional regulatory cascades in Runx2-dependent bone development. Tissue Eng. Part B Rev..

[21] Lv X., Gao F., Cao X. (2022). Skeletal interoception in bone homeostasis and pain. Cell Metab..

[22] Xu J., Zhang Z., Zhao J., Meyers C.A., Lee S., Qin Q., James A.W. (2022). Interaction between the nervous and skeletal systems. Front. Cell Dev. Biol..

[23] Chen H., Hu B., Lv X., Zhu S., Zhen G., Wan M., Jain A., Gao B., Chai Y., Yang M. (2019). Prostaglandin E2 mediates sensory nerve regulation of bone homeostasis. Nat. Commun..

[24] Gao F., Hu Q., Chen W., Li J., Qi C., Yan Y., Qian C., Wan M., Ficke J., Zheng J. (2024). Brain regulates weight bearing bone through PGE2 skeletal interoception: Implication of ankle osteoarthritis and pain. Bone Res..

[25] Thi M.M., Suadicani S.O., Schaffler M.B., Weinbaum S., Spray D.C. (2013). Mechanosensory responses of osteocytes to physiological forces occur along processes and not cell body and require αVβ3 integrin. Proc. Natl. Acad. Sci. USA.

[26] Turner C.H., Warden S.J., Bellido T., Plotkin L.I., Kumar N., Jasiuk I., Danzig J., Robling A.G. (2009). Mechanobiology of the skeleton. Sci. Signal..

[27] Chen Y.-H., Lo R.Y. (2017). Alzheimer’s disease and osteoporosis. Tzu Chi Med. J..

[28] Oughli H.A., Chen G., Philip Miller J., Nicol G., Butters M.A., Avidan M., Stark S., Lenze E.J. (2018). Cognitive Improvement in Older Adults in the Year After Hip Fracture: Implications for Brain Resilience in Advanced Aging. Am. J. Geriatr. Psychiatry.

[29] Birkner D., Pigorsch M., Riedlinger D., Möckel M., Lindner T., Schenk L., Deutschbein J. (2025). The vulnerability of hip fracture patients with cognitive impairment: An analysis of health conditions, hospital care, and outcomes. BMC Geriatr..

[30] Li Y., Xiao Y., Liu C. (2017). The Horizon of Materiobiology: A Perspective on Material-Guided Cell Behaviors and Tissue Engineering. Chem. Rev..

[31] Takayanagi H. (2007). Osteoimmunology: Shared mechanisms and crosstalk between the immune and bone systems. Nat. Rev. Immunol..

[32] Colonna M., Wang Y. (2016). TREM2 variants: New keys to decipher Alzheimer disease pathogenesis. Nat. Rev. Neurosci..

[33] Hayman A.R. (2008). Tartrate-resistant acid phosphatase (TRAP) and the osteoclast/immune cell dichotomy. Autoimmunity.

[34] Xing J., Titus A.R., Humphrey M.B. (2015). The TREM2-DAP12 signaling pathway in Nasu-Hakola disease: A molecular genetics perspective. Res. Rep. Biochem..

[35] Spangenberg E.E., Green K.N. (2017). Inflammation in Alzheimer’s disease: Lessons learned from microglia-depletion models. Brain Behav. Immun..

[36] Dallas S.L., Prideaux M., Bonewald L.F. (2013). The osteocyte: An endocrine cell … and more. Endocr. Rev..

[37] Youlten S.E., Kemp J.P., Logan J.G., Ghirardello E.J., Sergio C.M., Dack M.R., Guilfoyle S.E., Leitch V.D., Butterfield N.C., Komla-Ebri D. (2021). Osteocyte transcriptome mapping identifies a molecular landscape controlling skeletal homeostasis and susceptibility to skeletal disease. Nat. Commun..

[38] Delgado-Calle J., Sato A.Y., Bellido T. (2017). Role and mechanism of action of sclerostin in bone. Bone.

[39] Dobson P.F., Dennis E.P., Hipps D., Reeve A., Laude A., Bradshaw C., Stamp C., Smith A., Deehan D.J., Turnbull D.M. (2020). Mitochondrial dysfunction impairs osteogenesis, increases osteoclast activity, and accelerates age related bone loss. Sci. Rep..

[40] Elefteriou F. (2008). Regulation of bone remodeling by the central and peripheral nervous system. Arch. Biochem. Biophys..

[41] Méndez-Maldonado K., Vega-López G.A., Aybar M.J., Velasco I. (2020). Neurogenesis From Neural Crest Cells: Molecular Mechanisms in the Formation of Cranial Nerves and Ganglia. Front. Cell Dev. Biol..

[42] Komori T. (2008). Regulation of bone development and maintenance by Runx2. Front. Biosci..

[43] Khrimian L., Obri A., Ramos-Brossier M., Rousseaud A., Moriceau S., Nicot A.S., Mera P., Kosmidis S., Karnavas T., Saudou F. (2017). Gpr158 mediates osteocalcin’s regulation of cognition. J. Exp. Med..

[44] Lin J., Li Q., Lei X., Zhao H. (2022). The emerging roles of GPR158 in the regulation of the endocrine system. Front. Cell Dev. Biol..

[45] Li J., Lou S., Bian X. (2025). Osteocalcin and GPR158: Linking bone and brain function. Front. Cell Dev. Biol..

[46] Diegel C.R., Hann S., Ayturk U.M., Hu J.C.W., Lim K.E., Droscha C.J., Madaj Z.B., Foxa G.E., Izaguirre I., Transgenics Core V.V.A. (2020). An osteocalcin-deficient mouse strain without endocrine abnormalities. PLoS Genet..

[47] Knapen M.H., Eisenwiener H.G., Vermeer C. (1996). Osteocalcin detection in aging serum and whole blood: Stability of different osteocalcin fractions. Clin. Chim. Acta.

[48] Terreni A., Pezzati P. (2012). Biochemical markers in the follow-up of the osteoporotic patients. Clin. Cases Miner. Bone Metab..

[49] Kakonen S.-M., Hellman J., Karp M., Laaksonen P., Obrant K.J., Vaananen H.K., Lovgren T., Pettersson K. (2000). Development and evaluation of three immunofluorometric assays that measure different forms of osteocalcin in serum. Clin. Chem..

[50] Obri A., Khrimian L., Karsenty G., Oury F. (2018). Osteocalcin in the brain: From embryonic development to age-related decline in cognition. Nat. Rev. Endocrinol..

[51] Shan C., Ghosh A., Guo X.Z., Wang S.M., Hou Y.F., Li S.T., Liu J.M. (2019). Roles for osteocalcin in brain signalling: Implications in cognition- and motor-related disorders. Mol. Brain.

[52] Wang K.X., Denhardt D.T. (2008). Osteopontin: Role in immune regulation and stress responses. Cytokine Growth Factor Rev..

[53] Boggio E., Dianzani C., Gigliotti C.L., Soluri M.F., Clemente N., Cappellano G., Toth E., Raineri D., Ferrara B., Comi C. (2016). Thrombin Cleavage of Osteopontin Modulates Its Activities in Human Cells In Vitro and Mouse Experimental Autoimmune Encephalomyelitis In Vivo. J. Immunol. Res..

[54] Hansda S., Das H. (2025). Insights into Cancer-Associated Thrombosis Leading Towards Ischemic Stroke. Biology.

[55] Azizan Z., Bazrgar M., Bazgir N., Moini S.H., Ghaseminejad-Kermani S., Safa K., Eshaghian-Dorcheh A., Harirchian M.H. (2025). Osteopontin in Alzheimer’s Disease: A Double-Edged Sword in Neurodegeneration and Neuroprotection-A Systematic Review. CNS Neurosci. Ther..

[56] Castello L.M., Raineri D., Salmi L., Clemente N., Vaschetto R., Quaglia M., Garzaro M., Gentilli S., Navalesi P., Cantaluppi V. (2017). Osteopontin at the Crossroads of Inflammation and Tumor Progression. Mediat. Inflamm..

[57] Chabas D., Baranzini S.E., Mitchell D., Bernard C.C., Rittling S.R., Denhardt D.T., Sobel R.A., Lock C., Karpuj M., Pedotti R. (2001). The influence of the proinflammatory cytokine, osteopontin, on autoimmune demyelinating disease. Science.

[58] Comi C., Carecchio M., Chiocchetti A., Nicola S., Galimberti D., Fenoglio C., Cappellano G., Monaco F., Scarpini E., Dianzani U. (2010). Osteopontin is increased in the cerebrospinal fluid of patients with Alzheimer’s disease and its levels correlate with cognitive decline. J. Alzheimer’s Dis..

[59] Zwamborn R.A.J., Snijders C., An N., Thomson A., Rutten B.P.F., de Nijs L. (2018). Wnt Signaling in the Hippocampus in Relation to Neurogenesis, Neuroplasticity, Stress and Epigenetics. Prog. Mol. Biol. Transl. Sci..

[60] Tu X., Rhee Y., Condon K.W., Bivi N., Allen M.R., Dwyer D., Stolina M., Turner C.H., Robling A.G., Plotkin L.I. (2012). Sost downregulation and local Wnt signaling are required for the osteogenic response to mechanical loading. Bone.

[61] Fairfield H., Rosen C.J., Reagan M.R. (2017). Connecting Bone and Fat: The Potential Role for Sclerostin. Curr. Mol. Biol. Rep..

[62] Banks W.A., Sharma P., Bullock K.M., Hansen K.M., Ludwig N., Whiteside T.L. (2020). Transport of Extracellular Vesicles across the Blood-Brain Barrier: Brain Pharmacokinetics and Effects of Inflammation. Int. J. Mol. Sci..

[63] Abdelsalam M., Ahmed M., Osaid Z., Hamoudi R., Harati R. (2023). Insights into Exosome Transport through the Blood-Brain Barrier and the Potential Therapeutical Applications in Brain Diseases. Pharmaceuticals.

[64] Pulgar V.M. (2019). Transcytosis to cross the blood brain barrier, new advancements and challenges. Front. Neurosci..

[65] Gao M., Gao W., Papadimitriou J.M., Zhang C., Gao J., Zheng M. (2018). Exosomes-the enigmatic regulators of bone homeostasis. Bone Res..

[66] Lyu H., Xiao Y., Guo Q., Huang Y., Luo X. (2020). The Role of Bone-Derived Exosomes in Regulating Skeletal Metabolism and Extraosseous Diseases. Front. Cell Dev. Biol..

[67] Zhao C., Sun W., Zhang P., Ling S., Li Y., Zhao D., Peng J., Wang A., Li Q., Song J. (2015). miR-214 promotes osteoclastogenesis by targeting Pten/PI3k/Akt pathway. RNA Biol..

[68] Lee E.C., Choi D., Lee D.H., Oh J.S. (2025). Engineering Exosomes for CNS Disorders: Advances, Challenges, and Therapeutic Potential. Int. J. Mol. Sci..

[69] Sakellariou V.I., Grigoriou E., Mavrogenis A.F., Soucacos P.N., Papagelopoulos P.J. (2012). Heterotopic ossification following traumatic brain injury and spinal cord injury: Insight into the etiology and pathophysiology. J. Musculoskelet. Neuronal Interact..

[70] Bajwa N.M., Kesavan C., Mohan S. (2018). Long-term Consequences of Traumatic Brain Injury in Bone Metabolism. Front. Neurol..

[71] Zhou B.N., Zhang Q., Li M. (2023). Alzheimer’s disease and its associated risk of bone fractures: A narrative review. Front. Endocrinol..

[72] Yirmiya R., Bab I. (2009). Major depression is a risk factor for low bone mineral density: A meta-analysis. Biol. Psychiatry.

[73] Dantzer R., O’connor J.C., Freund G.G., Johnson R.W., Kelley K.W. (2008). From inflammation to sickness and depression: When the immune system subjugates the brain. Nat. Rev. Neurosci..

[74] Westbroek I., van der Plas A., de Rooij K.E., Klein-Nulend J., Nijweide P.J. (2001). Expression of serotonin receptors in bone. J. Biol. Chem..

[75] Paloneva J., Kestilä M., Wu J., Salminen A., Böhling T., Ruotsalainen V., Hakola P., Bakker A.B., Phillips J.H., Pekkarinen P. (2000). Loss-of-function mutations in TYROBP (DAP12) result in a presenile dementia with bone cysts. Nat. Genet..

[76] Jay T.R., von Saucken V.E., Landreth G.E. (2017). TREM2 in neurodegenerative diseases. Mol. Neurodegener..

[77] Franceschi C., Garagnani P., Vitale G., Capri M., Salvioli S. (2017). Inflammaging and ‘Garb-aging’. Trends Endocrinol. Metab..

[78] López-Otín C., Blasco M.A., Partridge L., Serrano M., Kroemer G. (2013). The hallmarks of aging. Cell.

[79] Marcantonio E.R., Saczynski J.S., Jones R.N. (2012). Cognitive trajectories after postoperative delirium. N. Engl. J. Med..

[80] Zhao Y., Chen H., Qiu F., He J., Chen J. (2023). Cognitive impairment and risks of osteoporosis: A systematic review and meta-analysis. Arch. Gerontol. Geriatr..

[81] Zhou R., Deng J., Zhang M., Zhou H.D., Wang Y.J. (2011). Association between bone mineral density and the risk of Alzheimer’s disease. J. Alzheimer’s Dis..

[82] Cotman C.W., Berchtold N.C., Christie L.A. (2007). Exercise builds brain health: Key roles of growth factor cascades and inflammation. Trends Neurosci..

[83] Farr J.N., Khosla S. (2019). Cellular senescence in bone. Bone.

[84] Hansda S., Ghosh G., Ghosh R. (2020). 9-phenyl acridine photosensitizes A375 cells to UVA radiation. Heliyon.

[85] Hansda S., Ghosh R. (2022). Bystander effect of ultraviolet A radiation protects A375 melanoma cells by induction of antioxidant defense. J. Environ. Sci. Health C Toxicol. Carcinog..

[86] Ghosh R., Hansda S. (2021). Targeted and non-targeted effects of radiation in mammalian cells: An overview. Arch. Biotechnol. Biomed..

[87] Li M.C.M., Chow S.K.H., Wong R.M.Y., Qin L., Cheung W.H. (2021). The role of osteocytes-specific molecular mechanism in regulation of mechanotransduction—A systematic review. J. Orthop. Transl..

[88] Stewart S., Darwood A., Masouros S., Higgins C., Ramasamy A. (2020). Mechanotransduction in osteogenesis. Bone Jt. Res..

[89] Deng A.F., Wang F.X., Wang S.C., Zhang Y.Z., Bai L., Su J.C. (2024). Bone-organ axes: Bidirectional crosstalk. Mil. Med. Res..

[90] Shi T., Shen S., Shi Y., Wang Q., Zhang G., Lin J., Chen J., Bai F., Zhang L., Wang Y. (2024). Osteocyte-derived sclerostin impairs cognitive function during ageing and Alzheimer’s disease progression. Nat. Metab..

[91] Rosić D., Budišin V., Vrabec-Matković D. (2014). Osteoporosis in patients with Parkinson’s disease. Reumatizam.

[92] Farr J.N., Xu M., Weivoda M.M., Monroe D.G., Fraser D.G., Onken J.L., Negley B.A., Sfeir J.G., Ogrodnik M.B., Hachfeld C.M. (2017). Targeting cellular senescence prevents age-related bone loss in mice. Nat. Med..

[93] Warden S.J., Hassett S.M., Bond J.L., Rydberg J., Grogg J.D., Hilles E.L., Bogenschutz E.D., Smith H.D., Fuchs R.K., Bliziotes M.M. (2010). Psychotropic drugs have contrasting skeletal effects that are independent of their effects on physical activity levels. Bone.

[94] Ohlsson C., Sjögren K. (2015). Effects of the gut microbiota on bone mass. Trends Endocrinol. Metab..

[95] Tyagi A.M., Darby T.M., Hsu E., Yu M., Pal S., Dar H., Li J.-Y., Adams J., Jones R.M., Pacifici R. (2021). The gut microbiota is a transmissible determinant of skeletal maturation. eLife.

[96] Krautkramer K.A., Fan J., Bäckhed F. (2021). Gut microbial metabolites as multi-kingdom intermediates. Nat. Rev. Microbiol..

[97] D’Amelio P., Sassi F. (2018). Gut Microbiota, Immune System, and Bone. Calcif. Tissue Int..

[98] Cryan J.F., O’Riordan K.J., Cowan C.S.M., Sandhu K.V., Bastiaanssen T.F.S., Boehme M., Codagnone M.G., Cussotto S., Fulling C., Golubeva A.V. (2019). The Microbiota-Gut-Brain Axis. Physiol. Rev..

[99] Duffuler P., Bhullar K.S., Wu J. (2024). Targeting gut microbiota in osteoporosis: Impact of the microbial-based functional food ingredients. Food Sci. Hum. Wellness.

[100] Zhang M.J., Pisco A.O., Darmanis S., Zou J. (2021). Mouse aging cell atlas analysis reveals global and cell type-specific aging signatures. eLife.

[101] Cai D., Khor S. (2019). “Hypothalamic microinflammation” paradigm in aging and metabolic diseases. Cell Metab..

[102] Kim Y.S., Joh T.H. (2006). Microglia, major player in the brain inflammation: Their roles in the pathogenesis of Parkinson’s disease. Exp. Mol. Med..

[103] Zhou R., Guo Q., Xiao Y., Guo Q., Huang Y., Li C., Luo X. (2021). Endocrine role of bone in the regulation of energy metabolism. Bone Res..

[104] Montagne A., Barnes S.R., Sweeney M.D., Halliday M.R., Sagare A.P., Zhao Z., Toga A.W., Jacobs R.E., Liu C.Y., Amezcua L. (2015). Blood-brain barrier breakdown in the aging human hippocampus. Neuron.

[105] Kusumbe A.P., Ramasamy S.K., Itkin T., Mäe M.A., Langen U.H., Betsholtz C., Lapidot T., Adams R.H. (2016). Age-dependent modulation of vascular niches for haematopoietic stem cells. Nature.

[106] Elefteriou F. (2018). Impact of the autonomic nervous system on the skeleton. Physiol. Rev..

[107] Yadav V.K., Ryu J.-H., Suda N., Tanaka K.F., Gingrich J.A., Schütz G., Glorieux F.H., Chiang C.Y., Zajac J.D., Insogna K.L. (2008). Lrp5 controls bone formation by inhibiting serotonin synthesis in the duodenum. Cell.

[108] Lewis J.W., Frost K., Neag G., Wahid M., Finlay M., Northall E.H., Abudu O., Kemble S., Davis E.T., Powell E. (2024). Therapeutic avenues in bone repair: Harnessing an anabolic osteopeptide, PEPITEM, to boost bone growth and prevent bone loss. Cell Rep. Med..

[109] Tomlinson R.E., Christiansen B.A., Giannone A.A., Genetos D.C. (2020). The Role of Nerves in Skeletal Development, Adaptation, and Aging. Front. Endocrinol..

[110] Park E.J., Truong V.L., Jeong W.S., Min W.K. (2024). Brain-Derived Neurotrophic Factor (BDNF) Enhances Osteogenesis and May Improve Bone Microarchitecture in an Ovariectomized Rat Model. Cells.

[111] Howlader M.S.I., Prateeksha P., Hansda S., Naidu P., Das M., Barthels D., Das H. (2024). Secretory products of DPSC mitigate inflammatory effects in microglial cells by targeting MAPK pathway. Biomed. Pharmacother..

[112] Prateeksha P., Howlader M.S.I., Hansda S., Naidu P., Das M., Abo-Aziza F., Das H. (2023). Secretome of dental pulp-derived stem cells reduces inflammation and proliferation of glioblastoma cells by deactivating Mapk-Akt pathway. Dis. Res..

[113] Zhang H.G., Grizzle W.E. (2014). Exosomes: A novel pathway of local and distant intercellular communication that facilitates the growth and metastasis of neoplastic lesions. Am. J. Pathol..

[114] Han W., Zhang H., Feng L., Dang R., Wang J., Cui C., Jiang P. (2023). The emerging role of exosomes in communication between the periphery and the central nervous system. MedComm.

[115] Han Y., Li X., Zhang Y., Han Y., Chang F., Ding J. (2019). Mesenchymal Stem Cells for Regenerative Medicine. Cells.

[116] Tian S., Zhou X., Zhang M., Cui L., Li B., Liu Y., Su R., Sun K., Hu Y., Yang F. (2022). Mesenchymal stem cell-derived exosomes protect against liver fibrosis via delivering miR-148a to target KLF6/STAT3 pathway in macrophages. Stem Cell Res. Ther..

[117] Jahangard Y., Monfared H., Moradi A., Zare M., Mirnajafi-Zadeh J., Mowla S.J. (2020). Therapeutic effects of transplanted exosomes containing miR-29b to a rat model of Alzheimer’s disease. Front. Neurosci..

[118] Arredondo S.B., Valenzuela-Bezanilla D., Santibanez S.H., Varela-Nallar L. (2022). Wnt signaling in the adult hippocampal neurogenic niche. Stem Cells.

[119] Bonilla C., Zurita M. (2021). Cell-Based Therapies for Traumatic Brain Injury: Therapeutic Treatments and Clinical Trials. Biomedicines.

[120] Yáñez-Mó M., Siljander P.R.-M., Andreu Z., Bedina Zavec A., Borràs F.E., Buzas E.I., Buzas K., Casal E., Cappello F., Carvalho J. (2015). Biological properties of extracellular vesicles and their physiological functions. J. Extracell. Vesicles.

[121] Wang D., Cao H., Hua W., Gao L., Yuan Y., Zhou X., Zeng Z. (2022). Mesenchymal stem cell-derived extracellular vesicles for bone defect repair. Membranes.

[122] Zhu H., Su Y., Wang J., Wu J.Y. (2025). Correction: The role of vesicle trafficking genes in osteoblast differentiation and function. Sci. Rep..

[123] Jiang Y.L., Wang Z.X., Liu X.X., Wan M.D., Liu Y.W., Jiao B., Liao X.X., Luo Z.W., Wang Y.Y., Hong C.G. (2022). The protective effects of osteocyte-derived extracellular vesicles against Alzheimer’s disease diminished with aging. Adv. Sci..

[124] Fang F., Yang J., Wang J., Li T., Wang E., Zhang D., Liu X., Zhou C. (2024). The role and applications of extracellular vesicles in osteoporosis. Bone Res..

[125] Hotamisligil G.S. (2006). Inflammation and metabolic disorders. Nature.

[126] De Felice F.G. (2013). Alzheimer’s disease and insulin resistance: Translating basic science into clinical applications. J. Clin. Investig..

[127] Ferron M., Wei J., Yoshizawa T., Del Fattore A., DePinho R.A., Teti A., Ducy P., Karsenty G. (2010). Insulin signaling in osteoblasts integrates bone remodeling and energy metabolism. Cell.

[128] Arnold S.E., Arvanitakis Z., Macauley-Rambach S.L., Koenig A.M., Wang H.-Y., Ahima R.S., Craft S., Gandy S., Buettner C., Stoeckel L.E. (2018). Brain insulin resistance in type 2 diabetes and Alzheimer disease: Concepts and conundrums. Nat. Rev. Neurol..

[129] Enriori P.J., Evans A.E., Sinnayah P., Jobst E.E., Tonelli-Lemos L., Billes S.K., Glavas M.M., Grayson B.E., Perello M., Nillni E.A. (2007). Diet-induced obesity causes severe but reversible leptin resistance in arcuate melanocortin neurons. Cell Metab..

[130] Li Y., Tian X., Luo J., Bao T., Wang S., Wu X. (2024). Molecular mechanisms of aging and anti-aging strategies. Cell Commun. Signal..

[131] Arron J.R., Choi Y. (2000). Bone versus immune system. Nature.

[132] Takayanagi H. (2012). New developments in osteoimmunology. Nat. Rev. Rheumatol..

[133] Yahara Y., Barrientos T., Tang Y.J., Puviindran V., Nadesan P., Zhang H., Gibson J.R., Gregory S.G., Diao Y., Xiang Y. (2020). Erythromyeloid progenitors give rise to a population of osteoclasts that contribute to bone homeostasis and repair. Nat. Cell Biol..

[134] Ciucci T., Ibáñez L., Boucoiran A., Birgy-Barelli E., Pène J., Abou-Ezzi G., Arab N., Rouleau M., Hébuterne X., Yssel H. (2015). Bone marrow Th17 TNFα cells induce osteoclast differentiation, and link bone destruction to IBD. Gut.

[135] Amarasekara D.S., Yun H., Kim S., Lee N., Kim H., Rho J. (2018). Regulation of osteoclast differentiation by cytokine networks. Immune Netw..

[136] Chen H., Shang D., Wen Y., Liang C. (2021). Bone-derived modulators that regulate brain function: Emerging therapeutic targets for neurological disorders. Front. Cell Dev. Biol..

[137] Yang N., Liu Y. (2021). The Role of the Immune Microenvironment in Bone Regeneration. Int. J. Med. Sci..

[138] Li Z., Gong C. (2025). NLRP3 inflammasome in Alzheimer’s disease: Molecular mechanisms and emerging therapies. Front. Immunol..

[139] Novack D.V. (2011). Role of NF-κB in the skeleton. Cell Res..

[140] Lin Q., Zhao B., Huang J., Chen R., Sun W., Ye Q., Yang L., Zhu X., Li X., Zhang R. (2025). Neuropeptides as regulators of bone metabolism: From molecular mechanisms to traditional Chinese medicine intervention strategies. Front. Pharmacol..

[141] Block M.L., Hong J.-S. (2005). Microglia and inflammation-mediated neurodegeneration: Multiple triggers with a common mechanism. Prog. Neurobiol..

[142] Ambrogini E., Almeida M., Martin-Millan M., Paik J.H., Depinho R.A., Han L., Goellner J., Weinstein R.S., Jilka R.L., O’Brien C.A. (2010). FoxO-mediated defense against oxidative stress in osteoblasts is indispensable for skeletal homeostasis in mice. Cell Metab..

[143] Long F., Schipani E., Asahara H., Kronenberg H., Montminy M. (2001). The CREB family of activators is required for endochondral bone development. Development.

[144] Inestrosa N.C., Varela-Nallar L. (2014). Wnt signaling in the nervous system and in Alzheimer’s disease. J. Mol. Cell Biol..

[145] Otto E., Knapstein P.-R., Jahn D., Appelt J., Frosch K.-H., Tsitsilonis S., Keller J. (2020). Crosstalk of brain and bone—Clinical observations and their molecular bases. Int. J. Mol. Sci..

[146] Das H., Kumar A., Lin Z., Patino W.D., Hwang P.M., Feinberg M.W., Majumder P.K., Jain M.K. (2006). Kruppel-like factor 2 (KLF2) regulates proinflammatory activation of monocytes. Proc. Natl. Acad. Sci. USA.

[147] Fisch S., Gray S., Heymans S., Haldar S.M., Wang B., Pfister O., Cui L., Kumar A., Lin Z., Sen-Banerjee S. (2007). Kruppel-like factor 15 is a regulator of cardiomyocyte hypertrophy. Proc. Natl. Acad. Sci. USA.

[148] Sen-Banerjee S., Mir S., Lin Z., Hamik A., Atkins G.B., Das H., Banerjee P., Kumar A., Jain M.K. (2005). Kruppel-like factor 2 as a novel mediator of statin effects in endothelial cells. Circulation.

[149] Jha P., Das H. (2017). KLF2 in regulation of NF-κB-mediated immune cell function and inflammation. Int. J. Mol. Sci..

[150] Laha D., Deb M., Das H. (2019). KLF2 (kruppel-like factor 2 [lung]) regulates osteoclastogenesis by modulating autophagy. Autophagy.

[151] Maity J., Deb M., Greene C., Das H. (2020). KLF2 regulates dental pulp-derived stem cell differentiation through the induction of mitophagy and altering mitochondrial metabolism. Redox Biol..

[152] Rolph D., Das H. (2020). Transcriptional regulation of osteoclastogenesis: The emerging role of KLF2. Front. Immunol..

[153] Ghaleb A.M., Yang V.W. (2017). Krüppel-like factor 4 (KLF4): What we currently know. Gene.

[154] Shi H., Sheng B., Zhang F., Wu C., Zhang R., Zhu J., Xu K., Kuang Y., Jameson S.C., Lin Z. (2013). Kruppel-like factor 2 protects against ischemic stroke by regulating endothelial blood brain barrier function. Am. J. Physiol. Heart Circ. Physiol..

[155] Chu H.-R., Sun Y.-C., Gao Y., Guan X.-M., Yan H., Cui X.-D., Zhang X.-Y., Li X., Li H., Cheng M. (2019). Function of Krüppel-like factor 2 in the shear stress-induced cell differentiation of endothelial progenitor cells to endothelial cells. Mol. Med. Rep..

[156] Barthels D., Prateeksha P., Nozohouri S., Villalba H., Zhang Y., Sharma S., Anderson S., Howlader M.S.I., Nambiar A., Abbruscato T.J. (2023). Dental pulp-derived stem cells preserve astrocyte health during induced gliosis by modulating mitochondrial activity and functions. Cell. Mol. Neurobiol..

[157] Laha D., Sarkar J., Maity J., Pramanik A., Howlader M.S.I., Barthels D., Das H. (2022). Polyphenolic compounds inhibit osteoclast differentiation while reducing autophagy through limiting ROS and the mitochondrial membrane potential. Biomolecules.

[158] Hansda S., Das S., Howlader M.S.I., Das M., Naidu P., Das B.C., Das H. (2025). Small Pharmacological Compound BT881 Inhibits Osteoclastic Differentiation by Limiting ROS and Modulating Mitochondrial Dysfunctions (Abstract ID: 158683). J. Pharmacol. Exp. Ther..

[159] Naidu P., Das M., Hansda S., Prateeksha P., Howlader M.S.I., Siraj M.A., Das H. (2025). Mechanisms of Ellagic Acid (EA)-Mediated Osteogenic Differentiation of Human Dental Pulp-Derived Stem Cells. ACS Omega.

[160] Parmar K.M. (2008). Kruppel-like Factor 2 as a Central Link Between Blood Flow and Vascular Endothelial Function.

[161] Cheng Z., Zou X., Jin Y., Gao S., Lv J., Li B., Cui R. (2018). The Role of KLF_4_ in Alzheimer’s Disease. Front. Cell. Neurosci..

[162] Wang H., Han J., Dmitrii G., Ning K., Zhang X.A. (2024). KLF transcription factors in bone diseases. J. Cell. Mol. Med..

[163] Kinisu M., Choi Y.J., Cattoglio C., Liu K., de Bezieux H.R., Valbuena R., Pum N., Dudoit S., Huang H., Xuan Z. (2021). Klf5 establishes bi-potential cell fate by dual regulation of ICM and TE specification genes. Cell Rep..

[164] Gao F., Hu Q., Qi C., Wan M., Ficke J., Zheng J., Cao X. (2023). Mechanical loading-induced change of bone homeostasis is mediated by PGE2-driven hypothalamic interoception. Res. Sq..

[165] Chiu S.H., Wu W.T., Yao T.K., Peng C.H., Yeh K.T. (2024). Sclerostin and Cardiovascular Risk: Evaluating the Cardiovascular Safety of Romosozumab in Osteoporosis Treatment. Biomedicines.

[166] Fabre S., Funck-Brentano T., Cohen-Solal M. (2020). Anti-sclerostin antibodies in osteoporosis and other bone diseases. J. Clin. Med..

[167] Zhao J., He Z., Wang J. (2021). MicroRNA-124: A key player in microglia-mediated inflammation in neurological diseases. Front. Cell. Neurosci..

[168] Takasugi M., Nonaka Y., Takemura K., Yoshida Y., Stein F., Schwarz J.J., Adachi J., Satoh J., Ito S., Tombline G. (2024). An atlas of the aging mouse proteome reveals the features of age-related post-transcriptional dysregulation. Nat. Commun..

[169] Nakamura M., Imaoka M., Takeda M. (2021). Interaction of bone and brain: Osteocalcin and cognition. Int. J. Neurosci..

[170] Schurman C.A., Burton J.B., Rose J., Ellerby L.M., Alliston T., Schilling B. (2023). Molecular and cellular crosstalk between bone and brain: Accessing bidirectional neural and musculoskeletal signaling during aging and disease. J. Bone Metab..

[171] Elefteriou F., Ahn J.D., Takeda S., Starbuck M., Yang X., Liu X., Kondo H., Richards W.G., Bannon T.W., Noda M. (2005). Leptin regulation of bone resorption by the sympathetic nervous system and CART. Nature.

[172] Zaidi M., Kim S.-M., Mathew M., Korkmaz F., Sultana F., Miyashita S., Gumerova A.A., Frolinger T., Moldavski O., Barak O. (2023). Bone circuitry and interorgan skeletal crosstalk. eLife.

[173] Abeynayake N., Arthur A., Gronthos S. (2021). Crosstalk between skeletal and neural tissues is critical for skeletal health. Bone.

[174] Chen G., Deng C., Li Y.-P. (2012). TGF-β and BMP signaling in osteoblast differentiation and bone formation. Int. J. Biol. Sci..

[175] Moustakas A., Heldin C.-H. (2009). The regulation of TGFβ signal transduction. Development.

[176] Itasaki N., Hoppler S. (2010). Crosstalk between Wnt and bone morphogenic protein signaling: A turbulent relationship. Dev. Dyn. Off. Publ. Am. Assoc. Anat..

[177] Mazzitelli J.A., Pulous F.E., Smyth L.C.D., Kaya Z., Rustenhoven J., Moskowitz M.A., Kipnis J., Nahrendorf M. (2023). Skull bone marrow channels as immune gateways to the central nervous system. Nat. Neurosci..

[178] Kiialainen A., Hovanes K., Paloneva J., Kopra O., Peltonen L. (2005). Dap12 and Trem2, molecules involved in innate immunity and neurodegeneration, are co-expressed in the CNS. Neurobiol. Dis..

[179] Turnbull I.R., Gilfillan S., Cella M., Aoshi T., Miller M., Piccio L., Hernandez M., Colonna M. (2006). Cutting edge: TREM-2 attenuates macrophage activation. J. Immunol..

[180] Bakalenko N., Kuznetsova E., Malashicheva A. (2024). The complex interplay of TGF-β and notch signaling in the pathogenesis of fibrosis. Int. J. Mol. Sci..

[181] Wu M.Y., Hill C.S. (2009). TGF-β superfamily signaling in embryonic development and homeostasis. Dev. Cell.

[182] Shi Y., Massagué J. (2003). Mechanisms of TGF-β signaling from cell membrane to the nucleus. Cell.

[183] Vo H.T. (2019). Modulation of Klotho Affects Dendritic Spine Remodeling and Neuronal Network Activity. Ph.D. Thesis.

[184] Maenhaut C., Christophe D., Vassart G., Dumont J., Roger P., Opitz R. (2015). Ontogeny, anatomy, metabolism and physiology of the thyroid. Endotext [Internet].

[185] Invernizzi M., Carda S., Viscontini G.S., Cisari C. (2009). Osteoporosis in Parkinson’s disease. Park. Relat. Disord..

[186] Cheng G., Huang C., Deng H., Wang H. (2012). Diabetes as a risk factor for dementia and mild cognitive impairment: A meta-analysis of longitudinal studies. Intern. Med. J..

[187] Huang C.-C., Chung C.-M., Leu H.-B., Lin L.-Y., Chiu C.-C., Hsu C.-Y., Chiang C.-H., Huang P.-H., Chen T.-J., Lin S.-J. (2014). Diabetes mellitus and the risk of Alzheimer’s disease: A nationwide population-based study. PLoS ONE.

[188] Hsu C.C., Wahlqvist M.L., Lee M.S., Tsai H.N. (2011). Incidence of dementia is increased in type 2 diabetes and reduced by the use of sulfonylureas and metformin. J. Alzheimer’s Dis..

[189] Su L., Liao Y., Liu X., Xie X., Li Y. (2023). Increased risk of dementia among people with a history of fractures: A systematic review and meta-analysis of population-based studies. Front. Neurol..

[190] Ioannidis I., Mohammad Ismail A., Forssten M.P., Ahl R., Cao Y., Borg T., Mohseni S. (2022). The mortality burden in patients with hip fractures and dementia. Eur. J. Trauma Emerg. Surg..

[191] Santos A., Bakker A.D., Klein-Nulend J. (2009). The role of osteocytes in bone mechanotransduction. Osteoporos. Int..

[192] Hiri O.T.P., Lawongsa K., Kengpanich S., Srisuwan P. (2024). Osteoporosis as a Potential Modifiable Risk Factor for Dementia in Thailand: A Cross-Sectional Analysis. Cureus.

[193] Li X., Wu X., Zhou G., Mo D., Lin X., Wang P., Zeng Y., Luo M. (2024). Estimated bone mineral density and white matter hyperintensities: A bidirectional Mendelian randomization study. Bone.

[194] Ilias I., Milionis C., Zoumakis E. (2022). An overview of glucocorticoid-induced osteoporosis. Endotext [Internet].

